# Organocatalytic kinetic resolution of 1,5-dicarbonyl compounds through a retro-Michael reaction

**DOI:** 10.3762/bjoc.21.34

**Published:** 2025-03-03

**Authors:** James Guevara-Pulido, Fernando González-Pérez, José M Andrés, Rafael Pedrosa

**Affiliations:** 1 Química Farmacéutica, Grupo Investigación en Química Aplicada INQA, Universidad El Bosque, Cra 7b Bis No 132 – 11, Bogotá, Colombia; 2 Instituto Universitario CINQUIMA y Departamento de Química Orgánica, Facultad de Ciencias, Universidad de Valladolid, Paseo Belén 7, 47011-Valladolid, Spain

**Keywords:** 1,5-dicarbonyl, equilibrium, kinetic resolution, organocatalysis, retro-Michael

## Abstract

The pharmaceutical chemical industry has long used kinetic resolution to obtain high-value compounds. Organocatalysis has recently been added to this strategy, allowing for the resolution of racemic mixtures with low catalyst loadings and mild reaction conditions. This research focuses on the kinetic resolution of 1,5-dicarbonyl compounds using a retro-Michael reaction, co-catalyzed at room temperature with 20 mol % of the Jørgensen–Hayashi catalyst and PNBA. The study highlights the importance of conducting the kinetic resolution at a concentration of approximately ten millimolar (mM) to prevent the Michael retro-Michael equilibrium from affecting the process.

## Introduction

For many years, enantiomers have been separated using chiral resolution. This involves separating the two enantiomers by converting the racemic mixture into a pair of diastereoisomers with the help of a chiral compound. The resulting diastereoisomers can be separated based on their physical properties using crystallization, distillation, or chromatography [1]. Sometime later, kinetic resolution (KR) emerged. This method is based on the different reaction rates of each enantiomer in a racemic mixture when they are reacted with a reagent, a chiral catalyst, or an enzyme. This process results in obtaining the less reactive enantioenriched enantiomer in the reaction mixture [2] and is the most practical method applied in the pharmaceutical industry [3]. However, research in this field has developed new resolution methods known as deracemization [4] and dynamic kinetic resolution (DKR) [5]. Currently, organocatalysis has enabled more efficient processes with low catalyst loading. It involves the kinetic resolution of alcohols, amines, and esters using chiral phosphoric acids [6–13] and sulfoximines with enals using chiral N-heterocyclic carbene (NHC) catalysts [14]. Additionally, these processes have been conducted using organometallic catalysis [15], enzymatic catalysis [16], aminocatalysis [17–19], and hydrogen-bonding catalysis [20–22].

**Scheme 1:**
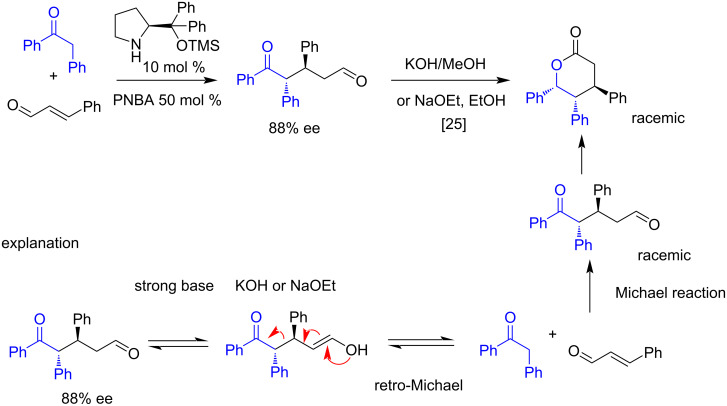
Previous work.

The Michael addition reaction is a versatile synthetic methodology that allows the formation of new carbon–carbon and carbon–heteroatom bonds through the coupling of electron-poor olefins with a wide range of nucleophiles, with many organocatalyzed asymmetric examples highlighted in the literature [23–24]. We have observed that the enantioenriched 1,5-dicarbonyl Michael adducts, synthesized via organocatalyzed reaction of cinnamaldehyde with benzyl phenyl ketone, undergo racemization when treated with inorganic bases [25], which had led us to check the equilibrium between Michael and the retro-Michael reaction (Scheme 1). These observations have prompted us to conduct further research into this reaction for potential applications in the kinetic resolution of these adducts.

In the literature, we found that 1,5-diketones and 1,5-ketoaldehydes have been utilized in retro-Michael reactions catalyzed by NaOH or KOH at extremely high reaction temperatures [26]. Some examples are also described under milder conditions, where the starting compounds are obtained with good chemical yields [27]. These reactions have been utilized in the enantioselective synthesis of aryl sulfoxides through the arylation of sulfonate anions in the presence of palladium catalysts [28–29]. They have also been used in the synthesis of the neuraminidase inhibitor (−)-oseltamivir [30] and the organocatalytic synthesis of 2-cyclohexen-1-ones via a Michael/Michael/retro-Michael cascade reaction [31].

**Scheme 2:**
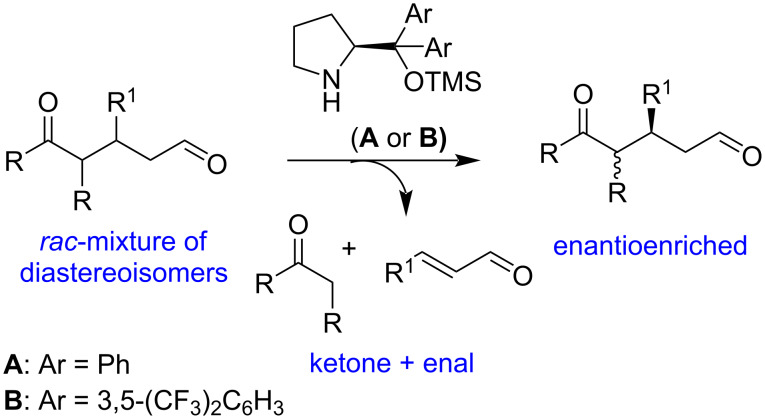
Hypothesis, retro-Michael reaction, and its application in kinetic resolution.

Our research has shown that the Jørgensen–Hayashi catalyst [32–33] is a highly promising organocatalyst, facilitating enantioselective Michael addition reactions with high yields and excellent levels of enantiocontrol [34–39]. In our studies on the organocatalytic enantioselective synthesis of 1,5-ketoaldehydes [40], we found that the prolinol derivative **A** is an outstanding catalyst for the enantioselective preparation of these adducts (Scheme 2). We are currently investigating whether this catalyst or the bistrifluoromethyl-substituted analog **B** could enable the retro-Michael reaction of only one enantiomer of the racemic mixture, potentially leading to a kinetic resolution of the 1,5-dicarbonyl compounds (Scheme 2).

## Results and Discussion

**Scheme 3:**
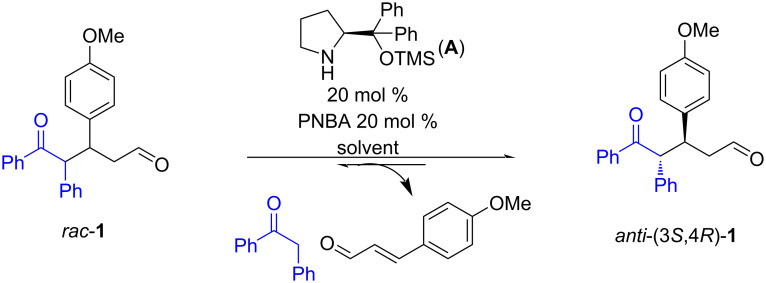
Model reaction.

An initial attempt was made to determine if the retro-Michael (ReM) reaction occurs, its enantio- and diastereoselectivity, and the influence of different experimental parameters on its scope and stereoselectivity. The reaction was studied on a 1:2 mixture of the racemic diastereoisomers *syn*-**1** and *anti*-**1** (prepared according to our previous protocol) [25] using 20 mol % of catalyst **A** and 20 mol % of *p*-nitrobenzoic acid (PNBA) as co-catalyst in different solvents at room temperature. The results obtained are summarized in Scheme 3 and Table 1.

**Table 1:** Solvent screening for the kinetic resolution of *rac*-**1**.

Entry	Solvent	Time (h)	er^a^	ReM (%)^b^

1	CH_2_Cl_2_	3	28:72	
2	CH_2_Cl_2_	5	22:78	
3	CH_2_Cl_2_	24	40:60	36
4	CHCl_3_	3	37:63	
5	CHCl_3_	5	35:65	
6	CHCl_3_	24	29:71	
7	CHCl_3_	72	35:65	33
8	Et_2_O	3	38:62	
9	Et_2_O	5	34:66	
10	Et_2_O	24	38:62	40
11	iPrOH	24	37:63	
12	iPrOH	72	44:56	25
13	MeOH	3	36:64	
14	MeOH	5	35:65	15
15	EtOH	24	38:62	
16	EtOH	72	33:67	15
17	TBME	24	33:67	
18	TBME	72	32:68	37
19	H_2_O	100	51:49	1
20	hexane	72	31:69	30
21	toluene	3	22:78	
22	toluene	5	19:81	
23	toluene	24	28:72	45

^a^er of the *anti*-diastereoisomer determined by chiral HPLC analysis. ^b^ReM (% of retro-Michael reaction) determined by ^1^H NMR.

The progress of the reaction was monitored using thin-layer chromatography (TLC) and ^1^H NMR analysis of the reaction mixture. The percentage of the retro-Michael reaction was calculated by comparing the signal of the starting 1,5-dicarbonyl adduct (*rac*-**1**) with the enal (cinnamaldehyde) product of the retro-Michael reaction. To determine whether the reaction favors one stereoisomer over the other and to assess its enantioselectivity, aliquots of the reaction mixture were taken at defined time intervals and analyzed by HPLC using a chiral column after passing them through a short silica gel pad.

Some interesting conclusions can be made from the data in Table 1. Firstly, the retro-Michael reaction occurs, to a greater or lesser extent, in all the solvents tested except in water (Table 1, entry 19), where the mixture remains unchanged after 100 hours. The enantiomeric ratio of the diastereomer *anti*-**1** depends on the solvent used, with toluene (Table 1, entry 22) providing the best results. Finally, enantioselectivity increased until a specific time, and after that, the enantiomeric ratio decreased (compare entries 1–3 and 21–23 in Table 1).

**Scheme 4:**
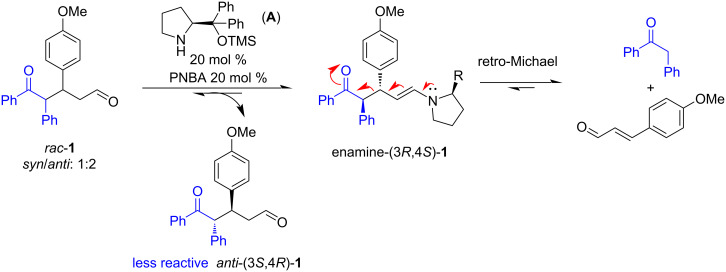
Kinetic resolution of the Michael adduct **1**.

The interesting result is that the major enantiomer in the enantioenriched mixture is now the opposite of the one obtained when the ketone and the α,β-unsaturated aldehyde are reacted in the presence of the catalyst **A** [25]. This can be explained by considering the principle of microscopic reversibility. In the reversible process, the catalyst forms the same enamine intermediate preferentially formed in the Michael reaction. This means that the adduct *anti*-(3*R*,4*S*)**-1** reacts more quickly than the *anti*-(3*S*,4*R*)-**1**, forming the enamine **E** (Scheme 4) that participates in the retro-Michael reaction, producing the starting ketone and the enal and enantio-enriching the reaction mixture in *anti*-(3*S*,4*R*)-**1**.

Subsequently, toluene was chosen as the solvent due to its ability to provide the highest enantiomeric ratio. The influence of catalyst, co-catalyst, and temperature on the reaction progress and enantioselectivity was further investigated. Different essays using 0.028 M toluene solutions were carried out, and the results are summarized in Table 2. The reaction also occurs without a co-catalyst but is slower, resulting in a lower enantiomeric ratio than in an acidic medium.

**Table 2:** Screening of catalyst and co-catalyst for kinetic resolution.

Entry	Catalyst (mol %)	Additive (mol %)	Temp.	Time (h)	ReM (%)^a^	er^b^

1	**A** (20)	–	rt	5	28	31:69
2	**A** (20)	PNBA (20)	rt	5	33	19:81
3	**A** (20)	PNBA (20)	rt	24	43	28:72
4	**A** (20)	BA (20)	rt	4	25	25:75
5	**A** (20)	BA (20)	rt	24	37	49:51
6	**B** (20)	PNBA (20)	rt	4	27	32:68
7	**B** (20)	PNBA (20)	rt	24	35	24:76
8	**B** (20)	BA (20)	rt	24	40	47:53
9	**A** (20)	K_2_CO_3_ (20)	rt	22	12	46:54
10	**A** (60)	PNBA (60)	rt	15	27	27:73
11	**A** (20)	PNBA (100)	rt	15	20	25:75
12	**A** (5)	PNBA (20)	rt	6	30	30:70
13	**A** (5)	PNBA (20)	rt	24	34	25:75
14	**A** (20)	PNBA (20)	−18 °C	100	0	50:50
15	**A** (20)	PNBA (20)	0 °C	6	25	35:65
16	**A** (20)	PNBA (20)	0 °C	24	27	33:67
17	**A** (20)	PNBA (20)	31 °C	0,16	14	43:57
18	**A** (20)	PNBA (20)	31 °C	0,33	38	31:69
19	**A** (20)	PNBA (20)	31 °C	0,66	44	28:72
20	**A** (20)	PNBA (20)	31 °C	3	30	35:65

^a^Determined by ^1^H NMR. ^b^Determined by chiral HPLC analysis.

The obtained results show that the diphenylprolinol derivative **A** provides a better enantiomeric ratio than that achieved with the α,α-bis[3,5-bis(trifluoromethyl)phenyl]prolinol derivative **B** (compare entry 2 versus entry 6 in Table 2). Furthermore, we studied the effect of using an organic acid as the co-catalyst for forming the enamine intermediate from **1** and for the retro-Michael reaction. We observed that benzoic acid (BA) as a co-catalyst provides a lower er than that achieved with PNBA as a co-catalyst (compare entries 2 and 3 with 4 and 5, or 7 with 8 in Table 2). In contrast, using a base as an additive slows the retro-Michel reaction and makes the reaction product a nearly racemic mixture (Table 2, entry 9).

Searching for the best reaction conditions, we varied the amounts of catalyst and additive (entries 10–13, Table 2), but none of the tests performed led to an improvement in enantioselectivity. We also studied the influence of the reaction temperature by performing two tests at 0 °C (entries 15 and 16, Table 2). We observed that the reaction occurs more slowly, and the enantiomeric excess reached is lower than at room temperature. Additionally, when the reaction mixture was stirred at −18 °C, no change was observed after 100 hours (entry 14, Table 2). These results led us to raise the reaction temperature to 31 °C (entries 17–20, Table 2). We observed that the retro-Michael reaction occurs more rapidly than at 20 °C (entry 2, Table 2). However, the enantiomeric ratio decreases as the reaction time increases.

Based on these results, we considered conducting tests to monitor how the percentage of ReM and enantiomeric ratio change over time (Table 3). With this aim, a 0.028 M mixture of racemic diastereomers **1** in toluene containing 20 mol % of catalyst **A** and 20 mol % of PNBA was stirred at room temperature. The data showed the highest enantiomeric ratio after four hours of reaction (entry 2, Table 3). However, it was also observed that when the retro-Michael process reached 50% extension, the enantiomer ratio decreased to approximately 1:2 (entry 4, Table 3). Furthermore, after 170 hours of reaction, the mixture became racemic, and the percentage of the retro-Michael process increased to 60% (entry 5, Table 3).

**Table 3:** Monitoring the kinetic resolution of **1** over time.

Entry	Time (h)	er^a^	ReM (%)^b^

1	0	50:50	0
2	4	16:84	31
3	24	28:72	43
4	47	36:64	50
5	170	49:51	60

^a^Determined by chiral HPLC analysis. ^b^Determined by ^1^H NMR.

These results can be explained by proposing that the catalyst initially promotes deracemization by rapidly reacting with the enantiomer (3*R*,4*S*) of the diastereomer *anti*-**1**. Over time, the initial equilibrium is established either because the catalyst begins to react with the *syn*-diastereomer or because, once the retro-Michael reaction has occurred, the catalyst promotes the Michael reaction, leading to the formation of the enantiomer (3*R*,4*S*) and consequently returning to the racemate.

Then, we decided to investigate how concentration affects the rate and selectivity of the reaction at room temperature (Table 4). The retro-Michael reaction mainly occurs at a concentration of 0.17 M, producing a nearly racemic mixture of *anti*-1 (entries 1 and 2, Table 4). Lowering the concentration to 0.10 M slows the reaction and improves the enantiomeric ratio (entries 3 and 4, Table 4), but a nearly racemic mixture is obtained again with longer reaction times (entry 5, Table 4). However, reducing the concentration to 0.014 M increases the enantioselectivity (entries 6 and 7, Table 4), and an excellent enantiomeric ratio was maintained over time (entries 8 and 9, Table 4). These results suggest that at very dilute concentrations, the decomposition of the enantiomer (3*R*,4*S*)-**1** is favored, preserving the (3*S*,4*R*)-**1** untransformed and avoiding the equilibrium reversal towards the formation of the Michael adduct, thereby preserving the enantiomeric purity of the isolated product.

**Table 4:** Study of the concentration's effect on kinetic resolution.

Entry	[M]	Time (h)	ReM (%)^a^	er^b^

1	0.17	14	40	45:55
2	0.17	38	55	46:54
3	0.10	28	30	40:60
4	0.10	46	45	38:62
5	0.10	117	58	48:52
6	0.014	9	32	16:84
7	0.014	32	35	14:86
8	0.014	48	40	8:92
9	0.014	60	40	8:92

^a^Determined by chiral HPLC analysis. ^b^Determined by ^1^H NMR.

To investigate the scope of the reaction, we extended our kinetic resolution study to a set of 5-functionalized aldehydes with at least one stereocenter in the C3 position. The results, which are collected in Table 5, show that the kinetic resolution depends on the characteristics of the substituents.

**Table 5:** Substrate the scope of kinetic resolution reactions.

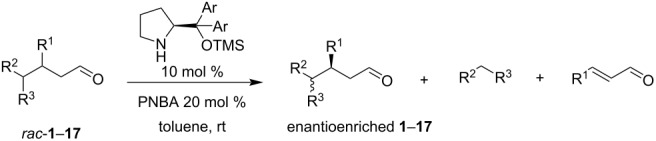											

Entry	Compound	R^1^	R^2^	R^3^	[mM]	*t* (h)	ReM^a^	Yield^b^	er^c^	dr^d^	dr^e^

1	**1**	*p*-MeOPh	COPh	Ph	14	48	40	50	8:92	1:2	1:2
2	**2**	*o*-NO_2_Ph	COPh	Ph	7	224	23	60	21:79	1:1	1:1
3	**3**	Ph	COPh	Ph	7	70	40	55	14:86	1:3	1:2
4	**4**	Ph	COEt	Ph	14	208	41	52	19:81	1:5	1:2
5	**5**	*p*-MeOPh	COEt	Ph	10	350	36	50	12:88	1:5	1:3
6	**6**	Ph	COBn	Ph	10	240	38	65	20:80	1:32	1:10
7	**7**	*p*-MeOPh	COBn	Ph	14	48	43	52	15:85	1:32	1:10
8	**8**	Me	COPh	SO_2_Ph	10	400	50	45	17:83	1:1	1:1
9	**9**	Et	COPh	SO_2_Ph	14	63	50	45	17:83	1:1	1:1
10	**10**	Ph	COMe	CO_2_Et	14	216	19	70	36:64	1:32	1:32
11	**11**	*p*-MeOPh	COMe	CO_2_Et	10	141	50	43	3:97^e^	1:32	1:32
12	**12**	Ph	COMe	COMe	8	144	50	40	6:94^f^	–	–
13	**13**	*p*-MeOPh	COMe	COMe	4	48	45	50	10:90	–	–
14	**14**	Ph	CO_2_Et	CO_2_Et	10	400	0	100	50:50	–	–
15	**15**	Ph	CO_2_Me	CO_2_Me	10	400	0	100	50:50	–	–
16	**16**	Ph	NO_2_	H	10	200	0	100	50:50	–	–
17	**17**	*p*-MeOPh	NO_2_	H	10	200	0	100	50:50	–	–

^a^% ReM (retro-Michael). ^b^Yield % refers to the total Michael adduct remaining unreacted. ^c^Chiral HPLC determined the enantiomeric ratio (er) for the major *anti*-diastereoisomer. ^d^initial dr: initial diastereomeric ratio (*syn*:*anti*). ^e^final dr: final diastereomeric ratio (*syn*:*anti*).

The best results were achieved for the Michael adduct **12**, derived from acetylacetone, with R^1^ = Ph (entry 12, Table 5), for the adduct **11**, derived from ethyl acetoacetate with R^1^ = *p*-(MeO)C_6_H_4_ (entry 11), and for **2** (entry 1). When comparing the enantiomeric ratio obtained for **12** with that obtained for the same compound when prepared by organocatalyzed Michael reaction [34], it is evident that the enantioselectivity is improved when prepared by the kinetic resolution method used in this work. The same improvement was also observed with product **2**. Additionally, it was possible to prepare enantioenriched product **11** (entry 11, Table 5), which had not been synthesized in an enantioselective manner until now.

For other Michael adducts prepared by reacting with differently activated ketones, the enantiomeric ratio of the isolated *anti*-diastereomer was excellent (entries 1 and 10–13, Table 5). Interestingly, the method also provides good resolution for Michael adducts **8** and **9** synthesized by reacting keto sulfones with enals having an aliphatic substituent in the β-position (entries 8 and 9, Table 5).

The resolution of Michael adducts **14** and **15** derived from dimethyl and diethyl malonate has also been studied (Table 5, entries 14 and 15). Unfortunately, the retro-Michael reaction did not take place under the conditions tested. Similarly, the resolution of nitro aldehydes **16** and **17**, prepared by Michael addition of nitromethane to α,β-unsaturated aldehydes (entries 16 and 17, Table 5), was tested, and the same results were obtained as in the cases of the malonate derivatives. These results indicate that dicarbonyl compounds are better leaving groups than diesters or nitro derivatives in this type of transformation. Another possible explanation is that Michael adducts other than 1,5-dicarbonyl compounds require more robust bases to shift the equilibrium toward the retro-Michael product. It is essential to highlight that the enantiomeric ratio values in Table 5 correspond to the major *anti*-diastereomer.

**Scheme 5:**
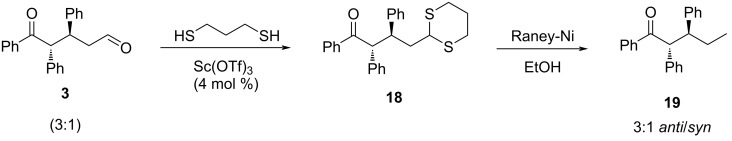
Chemical correlation of **3** with **19**.

The absolute configuration of the significant diastereoisomer obtained in the kinetic resolution of compound **3** was established by chemical correlation with (2*R*,3*S*)-1,2,3-triphenylpentan-1-one (**19**), previously described in the literature (Scheme 5) [41–42]. Treatment of a 3:1 mixture of the *anti/syn*-diastereoisomers of compound **3** with 1,3-propane dithiol in the presence of a small amount of scandium triflate [43] afforded compound **18**, which was used in the next step without further purification. Hydrogenolysis of **18** with Raney nickel in ethanol at room temperature gave a 3:1 mixture of *anti/syn*-**19**. The absolute configuration of *anti*-**19** is (2*R*,3*S*), indicating that in the resolution process, the major enantiomer corresponds to the *anti*-(3*S*,4*R*)-5-oxo-3,4,5-triphenylpentanal.

Having established that the major diastereomer of **1** is the *anti*-adduct, we attempted to study the behavior of the racemates of both diastereomers separately. We conducted additional experiments using racemic *anti*-**1** (entries 1–3 in Table 6). Unfortunately, we could not perform tests with pure racemic *syn*-**1** because it could only be isolated as a mixture with its epimer.

**Table 6:** Study of the kinetic resolution of pure diastereomer *anti*-**1**.

Entry	Time (h)	*syn*/*anti*^a^	er*_syn_*^b^	er*_anti_*^b^

1	5	1:13	4:96	30:70
2	9	1:7	6:94	20:80
3	48	1:7	20:80	16:84

^a^Determined by ^1^H NMR. ^b^Determined by chiral HPLC.

An epimerization process was observed when a 0.014 M solution of racemic *anti*-**1** in toluene was stirred at room temperature in the presence of the prolinol derivative **A** (20 mol %). This process led to a mixture of *syn/anti* epimers that changed over time and reached a ratio of 1:7 after 48 hours (entries 1–3 in Table 6). Our study determined the enantiomeric ratio of both *syn-* and *anti*-**1** diastereomers. After five hours, the er of the diastereomer *syn*-**1** was 4:96, decreasing to 20:80 after 48 h. In contrast, the er of the *anti*-diastereomer increased to 16:84 after 48 hours. This resulted in a slight decrease in enantioselectivity compared to the result obtained when starting from a 1:2 mixture of *syn/anti*-**1**.

**Scheme 6:**
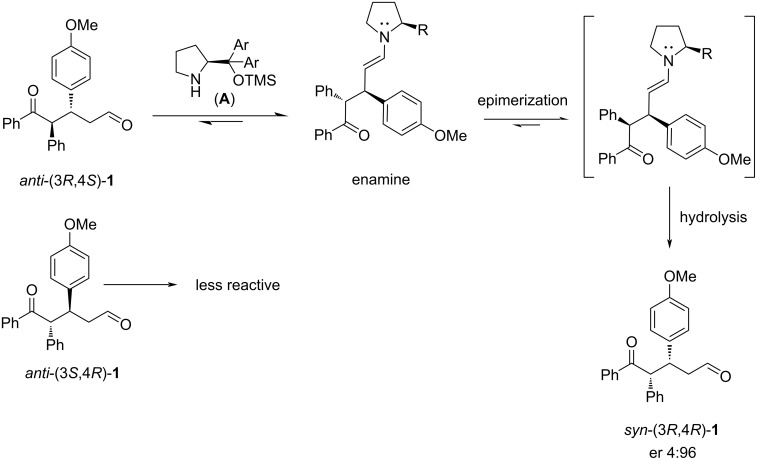
Epimerization of the *anti*-**1** adduct promoted by **A**.

Scheme 6 presents a possible explanation for the epimerization of the pure *rac*-*anti*-**1** adduct. The reaction of the more reactive enantiomer *anti*-(3*R*,4*S*)-**1** with catalyst **A** leads to enamine **E**, which epimerizes at C-4 and, after hydrolysis, provides the adduct *syn*-(3*R*,4*R*)-**1** with an initial er of 4:96. This diastereoselective epimerization phenomenon promoted by an organocatalyst has not been previously described.

The study emphasizes the reversibility of some organocatalyzed reactions and their impact on the enantioselectivity and diastereoselectivity of the products. The results show that Michael adducts can evolve from enantioenriched mixtures to racemic ones in the crude reaction while in contact with the chiral organocatalyst.

## Conclusion

The first example of the organocatalyzed kinetic resolution of 1,5-dicarbonyl compounds prepared by a Michael addition reaction has been described. The concentration of the reaction mixture significantly affects the retro-Michael process, achieving higher enantioselectivity in dilute solutions. The enantioselectivity also depends on the substituents present in the starting Michael adducts. Furthermore, it has been observed that the enantioselectivity of Michael adducts decreases with time in the presence of a catalyst derived from diarylprolinol.

## Experimental

### General Information

^1^H NMR (400 or 500 MHz) and ^13^C NMR (100 MHz) spectra were recorded in CDCl_3_ or acetone-*d*_6_. Chemical shifts for protons are reported in ppm from tetramethylsilane as an internal reference. Chemical shifts for carbons are reported in ppm from tetramethylsilane and referenced to the solvent's carbon resonance. Specific rotations were measured using a 5 mL cell with a one dm path length, and concentration was given in grams per 100 mL. TLC analysis was performed on glass-backed plates coated with silica gel 60 and an F254 indicator and visualized by either UV irradiation or staining with phosphomolybdic acid solution. Flash chromatography uses silica gel (230–240 mesh). Chiral HPLC analysis was performed using different chiral columns. IR spectra were recorded on an FTIR instrument. High-resolution mass spectra were performed by positive electrospray ionization using a quadrupole-time-of-flight detector (ESI+-Q-TOF) instrument. All compounds were purchased from commercial sources and used as received. Racemic compounds **14**–**17** were prepared by the general procedure as described, and their spectroscopic data agreed with the literature values [34,44].

**General procedure for the synthesis of racemic Michael adducts**. Racemic catalyst **A** (81 mg, 0.25 mmol) was added to a solution of enal (2.5 mmol) and 2-phenylacetophenone (3.0 mmol) in dichloromethane (20 mL), and the mixture was stirred until the reaction was completed. Then, the solvent was removed under reduced pressure, and the residue was purified by silica gel column chromatography using hexane/ethyl acetate mixtures as eluent, obtaining mixtures of racemic diastereoisomers **1**–**17** with yields of 56–70%.

**General procedure for kinetic resolution.** In a Wheaton flask, racemic Michael adducts **1**–**17** (0.42 mmol), *p*-nitrobenzoic acid (14 mg, 0.08 mmol), and catalyst **A** (27 mg, 0.08 mmol) were dissolved in toluene (30 mL). The mixture was stirred at room temperature for the necessary time (Table 5). Then, the reaction mixture was filtered through a short silica gel pack, and the solvent was evaporated under reduced pressure. The oily residue was purified on a silica gel chromatographic column using hexane/ethyl acetate mixtures as eluent.

**Experimental procedure for the chemical correlation of 3 with 19.** In a Wheaton flask, **3** (53 mg, 0.16 mmol), 1,3-propane dithiol (21 mg, 0.19 mmol, 1.2 equiv), and scandium triflate (3 mg, 0.0064 mmol, 0.04 equiv) in CH_2_Cl_2_ were mixed, and the resulting mixture was stirred for four hours at room temperature under argon atmosphere. Then, the solvent was removed under reduced pressure, and the reaction mixture was purified by silica gel column chromatography (eluent: hexane/ethyl acetate 4:1), obtaining **18** (57 mg, 0.136 mmol, 85%). Compound **18** (50 mg, 0.12 mmol) was dissolved in ethanol (5 mL), and Raney-Ni was added and stirred at room temperature for 12 h. Then, the mixture was filtered, the solid was washed four times with ethanol, and the resulting solution was concentrated under reduced pressure. The residue was purified by a silica gel chromatographic column (eluent: hexane/ethyl acetate 2:1), obtaining **19** (19 mg, 0.06 mmol, 50%).

## Supporting Information

File 1 Characterization data, copies of spectra and HPLC chromatograms.

## Data Availability

All data that supports the findings of this study is available in the published article and/or the supporting information of this article.
