# Supplementary material for: Organocatalytic kinetic resolution of 1,5-dicarbonyl compounds through a retro-Michael reaction
Source: Beilstein J Org Chem. 2025 Mar 3;21:473–82. doi: 10.3762/bjoc.21.34 (PMC11897654; doi:10.3762/bjoc.21.34)
Supplement: File 1 — Characterization data, copies of spectra and HPLC chromatograms. [file Beilstein_J_Org_Chem-21-473-s001.pdf]

BEILSTEIN JOURNAL OF ORGANIC CHEMISTRY

Supporting Information

for

Organocatalytic kinetic resolution of 1,5-dicarbonyl compounds through a retro-Michael reaction

James Guevara-Pulido, Fernando González-Pérez, José M. Andrés and Rafael Pedrosa

Beilstein J. Org. Chem. **2025,** *21,* 473–482. doi:10.3762/bjoc.21.34

Characterization data, copies of spectra and HPLC chromatograms

License and Terms: This is a supporting information file under the terms of the Creative Commons Attribution License (https://creativecommons.org/licenses/by/4.0). Please note that the reuse, redistribution and reproduction in particular requires that the author(s) and source are credited and that individual graphics may be subject to special legal provisions.
The license is subject to the *Beilstein Journal of Organic Chemistry* terms and conditions: (https://www.beilstein-journals.org/bjoc/terms)

Table of contents

1. Characterization data of compounds S2

2. References S7

2. NMR Spectra for new compounds S8

3. HPLC Chromatograms S22

1. Characterization data of compounds

***(S)*-3-(4-Methoxyphenyl)-5-oxo-4,5-diphenylpentanal** (**1**). White solid, mp 116-119 °C (hexane), (75 mg, 0.209 mmol, 50%), (84% ee). S*yn:anti* 1:2. ^{1}H-NMR (500 MHz, $CDCl_3$) δ 9.59 (dd, J = 2.8, 1.5 Hz, 0.3H, CHO_{syn}), 9.40 (dd, J = 2.5, 1.6 Hz, 0.7H, CHO_{anti}), 8.01 – 7.92 (m, 0.7H), 7.83 – 7.77 (m, 1.4H), 7.53 – 7.26 (m, 7H), 7.09 (td, J = 7.0, 2.9 Hz, 1.8H), 6.93 (d, J = 8.7 Hz, 1H), 6.80 – 6.76 (m, 1.4H), 6.67 (d, J = 8.7 Hz, 0.7H), 4.95 (d, J = 10.6 Hz, 0.7H, $CHPh_{anti}$), 4.80 (d, J = 10.5 Hz, 0.3H $CHPh_{syn}$), 4.22 (td, J = 10.2, 4.8 Hz, 0.7H, $CHC_6H_4OMe_{anti}$), 4.09 (td, J = 10.0, 4.6 Hz, 0.3H, $CHC_6H_4OMe_{syn}$), 3.73 (s, 2H, CH_3O_{anti}), 3.71 (s, 1H, CH_3O_{syn}), 2.93 – 2.78 (m, 0.6H), 2.62 – 2.48 (m, 1.4H). ^{13}C-NMR (126 MHz, $CDCl_3$) δ 201.3, 201.4, 199.4, 198.5, 158.4, 158.3, 137.0 136.8, 136.7, 134.1, 133.3, 132.9, 132.6, 129.4, 129.3, 129.1, 129.0, 128.9, 128.8, 128.7, 128.6, 128.5, 128.4, 128.0, 127.2, 114.1, 113.8, 59.74, 59.32, 55.22, 55.18, 48.25, 47.92, 43.31, 42.58. IR 2938, 1710, 1673, 1512, 1245, 695 cm^{-1}. HPLC (Chiralpak AD-H (hexane/iPrOH = 90:10) 1.0 mLmin^{-1} λ = 254 nm) t_R = 31.8 (minor), t_R = 33.2 (major). HRMS $C_{24}H_{23}O_3$ (M+H) Calc: 359.1642; Found: 359.1645.

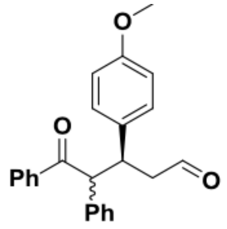

(*S*)-3-(2-Nitrophenyl)-5-oxo-4,5-diphenylpentanal (**2**). Yellow oil, (93 mg, 0.250 mmol, 60%), (58% ee). *Syn:anti* 1:2. ^{1}H-NMR (500 MHz, $CDCl_3$) δ 9.67 (s, 0.3H, CHO_{syn}), 9.45 (s, 0.7H, CHO_{anti}), 7.85 (dd, J = 68.9, 7.9 Hz, 3H), 7.30 (m, 11H), 5.16 (d, J = 10.1 Hz, 0.7H, $CHPh_{anti}$), 5.07 (d, J = 9.7 Hz, 0.3H, $CHPh_{syn}$), 4.85 (m, 0.3H $CHC_6H_4NO_{2syn}$), 4.75 (m, 0.7H $CHC_6H_4NO_2$*anti*), 3.03 (dd, J = 8.5, 2.7 Hz, 0.7H), 2.78 (dd, J = 16.9, 4.3 Hz, 0.7H), 2.60 (dd, J = 16.4, 8.1 Hz, 0.6H). ^{13}C-NMR (126 MHz, $CDCl_3$) δ 200.5, 197.4, 150.6, 150.5, 136.9, 136.5, 136.1, 135.9, 135.7, 135.4, 133.3, 133.1, 132.7, 132.4, 129.5, 129.0, 128.9, 128.7, 128.6, 128.5, 128.4, 128.3, 127.6, 127.5, 124.7, 124.4, 124.3, 58.4, 58.00, 47.46, 47.08, 29.66, 30.91. IR 3066, 3023, 1720, 1672, 1518, 1352, 701 cm^{-1}. HPLC (Chiralpak AD-H (hexane/iPrOH = 90:10) 0.5 mLmin^{-1} λ = 254 nm) t_R = 74.1 (minor), t_R = 77.4 (major). HRMS $C_{23}H_{20}NO_4$ (M+H) Calc: 374.1387; Found: 374.1390.

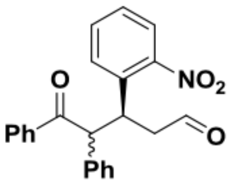

(*S*)-5-Oxo-3,4,5-triphenylpentanal (3).[1] White solid, mp 181-184 °C (hexane), (76 mg, 0.23 mmol, 55%), (72% ee). *Syn:anti* 1:3. ^{1}H-NMR (500 MHz, $CDCl_3$) δ (syn 9.59 (dd, J = 12.7, 10.6 Hz, 0.3), 9.39 (s, 0.7H), 8.13 – 7.91 (m, 1H), 7.78 (dd, J = 8.3, 1.0 Hz, 1.5H), 7.43 (dd, J = 12.5, 5.1 Hz, 2H), 7.42 – 7.38 (m, 1H), 7.38 – 7.30 (m, 3.5H), 7.30 – 7.27 (m, 1H), 7.29 – 7.17 (m, 3H), 7.12 (t, J = 7.4 Hz, 1H), 7.10 – 7.03 (m, 0.7H), 7.03 – 7.00 (m, 0.3H), 4.99 (d, J = 10.6 Hz, 0.7H, $CHPh_{anti}$), 4.83 (d, J = 10.5 Hz, 0.3H, $CHPh_{syn}$), 4.26 (td, J = 10.1, 4.9 Hz, 0.7H, CH_2CHPh_{anti}), 4.12 (d, J = 6.9 Hz, 0.3H, CH_2CHPh_{syn}), 2.63 (dd, J =

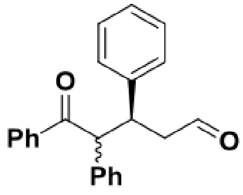

9.6, 2.5 Hz, 0.3H), 2.59 (dd, J = 9.6, 2.5 Hz, 0.7H), 2.54 (dd, J = 4.9, 1.5 Hz, 0.3H), 2.51 (dd, J = 4.9, 1.5 Hz, 0.7H). ^{13}C-NMR (126 MHz, $CDCl_3$) δ 201.2, 201.0, 198.4, 142.1, 137.0, 136.6, 133.3, 132.9, 129.4, 129.1, 128.9, 128.5, 128.4, 128.1, 127.2, 127.0, 126.8, 59.61, 59.07, 48.17, 47.86, 44.09, 43.38. IR 3060, 3034, 1713, 1671, 1499, 1447, 695 cm^{-1}. HPLC (Chiralpak AD-H (hexane/iPrOH = 95:5) 1.0 mLmin^{-1} λ = 254 nm) t_R = 31.6 (minor), t_R = 33.1 (major). HRMS $C_{23}H_{21}O_2$ (M+H) Calc: 329.1536; Found: 329.1540.

(3*S*,4*R*)-5-Oxo-3,4-diphenylheptanal (**4**).[2] White solid, mp 95-98 °C (hexane), (61 mg, 0.217 mmol, 52%). $[\alpha]_D^{25}$ = + 9.2 (c = 1, $CHCl_3$, 62% ee). ^{1}H- NMR (500 MHz, $CDCl_3$) δ 9.37 (dd, J = 2.5, 1.6 Hz, 1H), 7.37 (s, 1H), 7.36 (s, 2H), 7.33 (d, J = 4.4 Hz, 1H), 7.32 – 7.29 (m, 4H), 7.27 (d, J = 0.5 Hz, 1H), 7.21 (dt, J = 5.5, 4.0 Hz, 1H), 4.08 – 4.03 (m, 2H), 2.60 – 2.52 (m, 1H), 2.44 – 2.37 (m, 1H), 2.35 – 2.28 (m, 1H), 2.02 – 1.95 (m, 1H), 0.72 (t, J = 7.3 Hz, 3H); ^{13}C-NMR (126 MHz, $CDCl_3$) δ 209.3, 200.8, 141.8, 136.2, 129.2, 128.9, 128.8, 128.7, 128.5, 128.3, 128.2, 128.1, 128.0, 127.0, 64.13, 47.57, 42.62, 36.70, 7.38. IR 3034, 2938, 1710, 1496, 1454, 696 cm^{-1}. HPLC (Chiralcel OJ-H (hexane/iPrOH = 95:5) 1.0 mLmin^{-1} λ = 220 nm) t_R = 24.3 (major); t_R = 27.4 (minor). HRMS $C_{19}H_{21}O_2$ (M+H), Calc: 281.1536, Found: 281.1536.

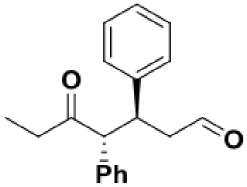

(3*S*,4*R*)-3-(4-Methoxyphenyl)-5-oxo-4-phenylheptanal (**5**). White solid, mp 100-109 °C (hexane), (65 mg, 0.209 mmol, 50%). $[\alpha]_D^{20}$ = + 6.2 (c = 1, $CHCl_3$, 76% ee). ^{1}H-NMR (500 MHz, $CDCl_3$) δ 9.34 (s, 1H), 7.41 (d, J = 15.8 Hz, 2H), 7.35 (s, 1H), 7.21 (d, J = 8.3 Hz, 2H), 6.82 (d, J = 8.3 Hz, 2H), 6.59 (dd, J = 15.8, 7.7 Hz, 2H), 4.00 (d, J = 2.9 Hz, 2H), 3.75 (s, 3H), 2.50 (dd, J = 10.8, 7.8 Hz, 1H), 2.38 – 2.26 (m, 2H), 2.03 – 1.94 (m, 1H), 0.71 (t, J = 7.1 Hz, 3H); ^{13}C-NMR (126 MHz, $CDCl_3$) δ 209.5, 201.1, 162.2, 158.4, 152.7, 136.4, 133.7, 129.1, 129.0, 128.8, 128.0, 126.8, 126.6, 114.1, 64.37, 55.19, 47.63, 41.87, 36.77, 7.41. IR 2943, 2842, 1710, 1662, 1598, 1507, 1254, 824 cm^{-1}. HPLC (Chiralpak AD-H (hexane/iPrOH = 90:10) 1.0 mLmin^{-1} λ = 254 nm) t_R = 12.1 (major); t_R = 14.2 (minor). HRMS $C_{20}H_{23}O_3$ (M+H) Calc: 311.1642; Found: 311.1638.

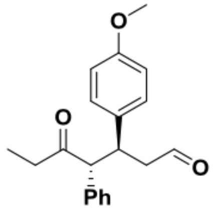

(3*S*,4*R*)-5-Oxo-3,4,6-triphenylhexanal (**6**).[2] White solid, mp 119-121 °C (hexane), (93 mg, 0.272 mmol, 65%). $[\alpha]_D^{20}$ = - 68.1 (c = 1, $CHCl_3$, 62% ee). ^{1}H-NMR (500 MHz, $CDCl_3$) δ 9.33 (s, 1H), 7.55 – 7.17 (m, 13H), 6.71 (d, J = 6.3 Hz, 2H), 4.13 (d, J = 11.1 Hz, 1H), 4.05 (dd, J = 10.1, 4.1 Hz, 1H), 3.38 (q, J = 15.7 Hz, 2H), 2.50 (dd, J = 14.8, 10.2 Hz, 1H), 2.35 (dd, J = 16.9, 3.0 Hz, 1H); ^{13}C-NMR (126 MHz, $CDCl_3$) δ 205.7, 200.7, 2C (141.5), 2C(135.8), 2C(133.2), 2C(129.5), 129.2, 129.0, 128.7, 128.6, 128.5, 128.1, 128.1,

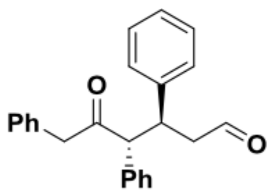

127.1, 126.9, 126.8, 62.75, 50.23, 47.51, 42.60. IR 3055, 3028, 1704, 1491, 1454, 701 cm^{-1}. HPLC (Chiralpak AD-H (hexane/iPrOH = 97:3) 0.7 $mLmin^{-1}$ λ = 220 nm) t_R = 35.4 (minor); t_R = 37.8 (major). HRMS $C_{24}H_{23}O_2$ (M+H), Calc: 343.1693, Found: 343.1 692.

(3*S*,4*R*)-3-(4-Methoxyphenyl)-5-oxo-4,6-diphenylhexanal (**7**).[2] White solid, mp 83-85 °C (hexane), (81 mg, 0.217 mmol, 52%). $[\alpha]_D^{20}$ = - 66.4 (c = 1, CH_2Cl_2, 70% ee). ^{1}H-NMR (500 MHz, $CDCl_3$) δ 9.32 (dd, J = 2.4, 1.3 Hz, 1H), 7.37 – 7.34 (m, 2H), 7.31 – 7.29 (m, 2H), 7.19 – 7.15 (m, 4H), 7.09 (d, J = 8.7 Hz, 2H), 6.78 (d, J = 8.6 Hz, 2H), 6.75 – 6.72 (m, 2H), 4.09 (d, J = 11.1 Hz, 1H), 4.01 (dd, J = 10.5, 4.5 Hz, 1H), 3.79 (s, 3H), 3.38 (d, J = 6.6 Hz, 2H), 2.46 (ddd, J = 16.7, 10.2, 2.7 Hz, 1H), 2.32 (ddd, J = 16.7, 4.4, 1.2 Hz, 1H); 13C-NMR (126 MHz, CDCl3) δ 205.9, 201.0, 158.4, 2C(135.9), 2C(133.4), 2C(133.2), 129.5, 129.4, 129.2, 129.1, 129.0, 128.7, 128.5, 128.1, 127.1, 126.8, 114.0, 63.03, 55.22, 50.30, 47.55, 41.84. IR 3023, 2906, 1715, 1512, 1496, 1251, 696 cm^{-1}. HPLC (Chiralpak AD-H (hexane/iPrOH = 90:10) 1.0 $mLmin^{-1}$ λ = 220 nm) t_R = 16.4 (major); t_R = 21.2 (minor). HRMS $C_{25}H_{25}O_3$ (M+H), Calc: 373.1798, Found: 373.1798.

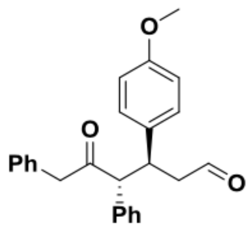

(3*S*)-3-Methyl-5-oxo-5-phenyl-4-(phenylsulfonyl)pentanal (**8**).[3] Yellow oil, (63 mg, 0.19 mmol, 45%), $[\alpha]_D^{20}$ = - 8.25 (c = 1, CH_2Cl_2, 66% ee). *Syn:anti* 1:1. ^{1}H-NMR (500 MHz, $CDCl_3$) δ 9.82 (s, 0.5H, CHO_{anti}), 9.67 (d, J = 0.7 Hz, 0.5H, CHO_{syn}), 7.58 (m, 10H), 5.50 (d, J = 9.4 Hz, 0.5H), 5.30 (d, J = 0.8 Hz, 0.5H), 3.06 (m, 2.5H), 2.49 (m, 0.5H), 1.29 (d, J = 7.0 Hz, 1.5H), 1.05 (d, J = 6.9 Hz, 1.5H). ^{13}C-NMR (126 MHz, $CDCl_3$) δ 200.6, 200.5, 193.2, 193.1, 138.1, 137.7, 137.0, 137.2, 134.1, 134.0, 133.9, 129.5, 129.4, 129.0, 128.9, 128.8, 128.7, 128.6, 128.5, 72.5, 71.6, 47.4, 47.3, 29.1, 28.7, 18.9, 18.5. IR 3065, 2932, 1720, 1677, 1309, 1149, 743 cm^{-1}. HPLC (Chiralpak AD-H (hexane/iPrOH = 40:60) 0.5 $mLmin^{-1}$ λ = 220 nm) t_R = 16.8 (minor); t_R = 18.4 (major). HRMS $C_{18}H_{19}SO_4$ (M+H) Calc: 331.0999; Found: 331.1000.

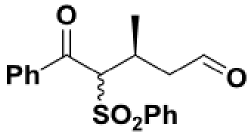

(3*S*)-3-Ethyl-5-oxo-5-phenyl-4-(phenylsulfonyl)pentanal (**9**).[4] Yellow oil (65 mg, 0.19 mmol, 45%), ($[\alpha]_D^{20}$ = - 7.25 (c = 1, CH_2Cl_2,66% ee). *Syn:anti* 1:1. ^{1}H-NMR (500 MHz, $CDCl_3$) δ 9.85 (s, 0.5H), 9.71 (s, 0.5H), 7.60 (m, 10H), 5.61 (d, J = 9.4 Hz, 0.5H), 5.36 (d, J = 5.0 Hz, 0.5H), 3.24 (ddd, J = 19.1, 10.0, 4.8 Hz, 1H), 3.10 (dd, J = 19.1, 3.8 Hz, 0.5H), 2.92 (m, 0.5H), 2.82 (d, J = 5.3 Hz, 0.5H), 2.52 (dd, J = 18.9, 7.8 Hz, 0.5H), 1.52 (m, 2H), 0.88 (t, J = 7.4 Hz, 1.5H), 0.82 (t, J = 7.4 Hz, 1.5H); ^{13}C-NMR (126 MHz, $CDCl_3$) δ 201.0, 200.8, 193.3, 193.0, 138.3, 137.9, 137.3, 137.1, 134.2, 134.1, 133.9, 134.0, 129.5, 129.3, 129.1, 128.9, 128.8, 128.7, 128.5, 128.4, 70.76, 70.09, 44.55, 42.82, 35.38, 34.52, 25.65, 24.29, 11.50,

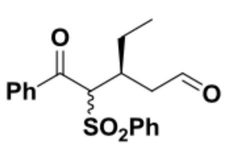

11.27. IR 3066, 2938, 1720, 1678, 1310, 1149, 744 cm^{-1}. HPLC (Chiralpak AD-H (hexane/iPrOH = 40:60) 0.5 $mLmin^{-1}$ λ = 220 nm) t_R = 60.6 (major) t_R = 66.4 (minor) HRMS $C_{19}H_{21}SO_4$ (M+H) Calc: 345.1155; Found: 345.1156.

(2*S*,3*S*)-Ethyl-2-acetyl-5-oxo-3-phenylpentanoate (**10**).[5] Yellow oil, (77 mg, 0.294 mmol, 70%). $[\alpha]_D^{20}$ = + 8.5 (c = 1, CH_2Cl_2, 28% ee). ^{1}H-NMR (500 MHz, $CDCl_3$) δ 9.54 (m, 1H), 7.22 (m, 5H), 3.99 (m, 2H), 3.89 (dd, J = 3.4, 2.8 Hz, 1H), 3.86 (d, J = 0.7 Hz, 1H), 2.80 (m, 1H), 2.75 (m, 1H), 2.22 (d, J = 0.5 Hz, 3H), 0.93 (td, J = 7.1, 0.7 Hz, 3H). ^{13}C-NMR (126 MHz, $CDCl_3$) δ 201.8, 200.3, 167.6, 140.0, 128.6, 128.3, 128.1, 128.0, 127.4, 65.28, 61.46, 47.57, 38.95, 29.76, 13.65. IR 2975, 2927, 1710, 1619, 1368, 1272, 1074, 1016, 760, 701 cm^{-1}. HPLC (Chiralpak AD-H (hexane/iPrOH = 95:5) 1.0 $mLmin^{-1}$ λ = 254 nm) t_R = 15.6 (major); t_R = 17.7 (minor). HRMS $C_{15}H_{19}O_4$ (M+H) Calc: 263.1278; Found: 263.1279.

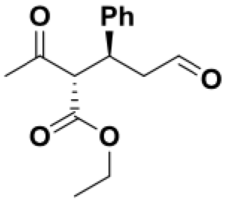

Ethyl (2*S*,3*S*)-2-acetyl-3-(4-methoxyphenyl)-5-oxopentanoate (**11**). Yellow oil, (53 mg, 0.18 mmol, 43%). $[\alpha]_D^{20}$ = + 19.9 (c = 1, CH_2Cl_2, 94% ee). ^{1}H-NMR (400 MHz, $CDCl_3$) δ 9.63 – 9.50 (m, 1H), 7.15 (ddd, J = 6.3, 4.7, 2.8 Hz, 2H), 6.84 – 6.80 (m, 2H), 4.29 – 4.10 (m, 2H), 3.95 – 3.92 (m, 1H), 3.87 (dd, J = 10.4, 2.9 Hz, 1H), 3.77 (d, J = 2.0 Hz, 3H), 2.85 – 2.78 (m, 1H), 2.78 – 2.72 (m, 1H), 2.33 – 2.17 (m, 3H), 1.27 (s, 3H); ^{13}C-NMR (126 MHz, $CDCl_3$) δ 201.9, 200.5, 167.6, 158.7, 131.9, 129.1, 129.1, 128.4, 114.0, 65.53, 61.45, 55.17, 47.68, 38.24, 29.78, 13.75. IR 2932, 2836, 1720, 1614, 1512, 1283, 1245, 1176, 1021, 834, 733 cm^{-1}. HPLC (Chiralpak AD-H (hexane/iPrOH = 95:5) 1.0 $mLmin^{-1}$ λ = 254 nm) t_R = 23.0 (minor); t_R = 25.9 (major). HRMS $C_{16}H_{21}O_5$ (M+H) Calc: 293.1384; Found: 293.1381.

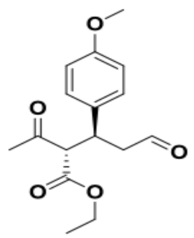

(*S*)-4-Acetyl-5-oxo-3-phenylhexanal (**12**).[6] White solid, mp 88-87 ˚C (hexane), (39 mg, 0.168 mmol, 40%). $[\alpha]_D^{25}$ = + 23.1 (c = 0.6, $CHCl_3$, 89% ee). ^{1}H-NMR (500 MHz, $CDCl_3$) δ 9.56 (dd, J = 2.1, 1.3 Hz, 1H), 7.26 (m, 5H), 4.20 (d, J = 11.0 Hz, 1H), 4.05 (s, 1H), 2.71 (m, 2H), 2.23 (s, 3H), 1.87 (s, 3H). ^{13}C-NMR (126 MHz, $CDCl_3$) δ 30.0, 30.3, 40.0, 48.0, 74.4, 127.8, 2C(128.0), 2C(129.2), 139.8, 200.1, 202.6, 202.9 IR. 3394, 3029, 2836, 2737, 1682, 1358, 1165, 706 cm^{-1} HPLC (Chiralcel OJ-H hexane/iPrOH 90 = 10; 1.0 mL/min λ = 220nm); t_R = 16.4 min (minor); t_R = 20.8.min (major). HRMS $C_{14}H_{17}O_3$ (M+H) Calc: 233.1172; Found: 233.1169.

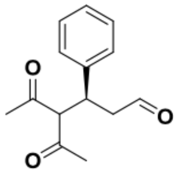

(*S*)-4-Acetyl-3-(4-methoxyphenyl)-5-oxohexanal compound with 1-((4*R*)-2-hydroxy-4-(4-methoxyphenyl)-6-methyl-3,4-dihydro-2*H*-pyran-5-yl)ethanone

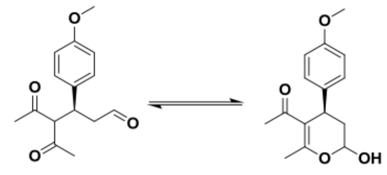

(**13**). White solid, mp 77-80 °C (hexane), (55 mg, 0.21 mmol, 50%). $[\alpha]_D^{25}$ = + 42.0 (c = 0.6, $CHCl_3$, 80% ee). ^{1}H-NMR (500 MHz, $CDCl_3$) δ 9.54 (m, 0.5H), 7.09 (m, 2H), 6.82 (m, 2H), 5.13 (d, J = 8.2 Hz, 0.5H), 4.92 (m, 0.5H), 4.47 (s, 1H), 4.15 (d, J = 11.0 Hz, 0.5H), 4.00 (m, 1H), 3.77 (m, 2H), 3.24 (m, 0.5H), 2.90 (d, J = 11.9 Hz, 0.5H), 2.66 (ddd, J = 6.9, 6.1, 1.8 Hz, 1H), 2.25 (m, 3H), 1.86 (t, J = 55.1 Hz, 3H). ^{13}C-NMR (126 MHz, $CDCl_3$) δ 214.6, 202.9, 202.8 202.0, 200.3, 159.0, 133.3, 131.6, 129.2, 128.8, 128.4, 114.6, 114.3, 95.4, 74.5, 68.8, 62.3 55.3, 48.1, 41.1, 39.1, 37.5, 36.2, 33.2, 31.0, 30.3, 29.9, 29.0, 28.6, 20.8. IR 3449, 2993, 2929, 2834, 2745, 1684, 1610, 1511 cm^{-1}. HPLC (Chiralcel OJ-H hexane/iPrOH 90 = 10; 1.0 mL/min λ = 220nm); t_R = 20.7 min (minor); t_R = 24.6.min (major). HRMS $C_{15}H_{18}O_4Na$ (M+Na) Calc: 285.1097; Found: 285.1098.

(2*R*,3*S*)-1,2,3-triphenylpentan-1-one (19).[7] White solid, mp 166-168 °C (hexane).

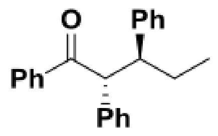

$[\alpha]D^{25}$ = - 8.8 (c = 1, CH_2Cl_2), (21 mg, 0.068 mmol, 50%). ^{1}H-NMR (500 MHz, $CDCl_3$) δ 7.29 (m, 15H), 4.84 (d, J = 10.9 Hz, 1H), 3.39 (td, J = 10.6, 4.2 Hz, 1H), 1.35 (m, 2H), 0.53 (t, J = 7.3 Hz, 3H). ^{13}C-NMR (126 MHz, $CDCl_3$) δ 199.6, 143.5, 2C(138.0), 137.6, 132.6, 129.2, 129.0, 128.8, 128.7, 128.5, 128.4, 128.3, 128.35, 127.4, 126.3, 60.0, 50.5, 26.5, 11.8.IR 3055, 2960, 2925, 2855, 1675, 1540, 1400, 695 cm-1. HPLC (Chiralpak AD-H hexano/iPrOH 97 = 3; 0.7 mL/min λ = 254nm); t_R = 13.4 min (minor); t_R = 14.7.min (major).

2. References

[1] Guevara-Pulido, J.; Andrés, J. M.; Pedrosa, R. *J. Org. Chem.* **2014**, *79*, 8638-8644.

[2] Guevara-Pulido, J.; Andrés, J. M.; Pedrosa. R. *RSC Advances* **2015**, *5*, 65975-65981.

[3] Enders, D.; Grossmann, A.; Huang, H.; Raabe, G. *Eur. J. Org. Chem.* **2011**, 4298-4301.

[4] Arpa, E. M.; Frias, M.; Alvarado, C.; Aleman, J.; Diaz-Tendero, S. *J. Mol. Catal. A Chem*. **2016**, *423*, 308-318.

[5] Guo, Y.; Xia, A.; Meng, C.; Xu, Z.; Xu, D.. Green Chem. **2017**,*19*, 2546-2549.

[6] Blanco, V.; Leigh, D. A.; Lewandowska, U.; Lewandowski, B.; Marcos, V. *J. Am. Chem. Soc.* **2014**, *136*, 15775-15780.

[7] (a) Auerbach, R. A.; Kingsbury, C. A. *Tetrahedron* **1973**, *29*, 1457-1464. (b) Zimmernan, H. E.; Chang, W-H. *J. Am. Chem. Soc.* **1958**, *81*, 3634-3643.

2. NMR Spectra for new compounds

1

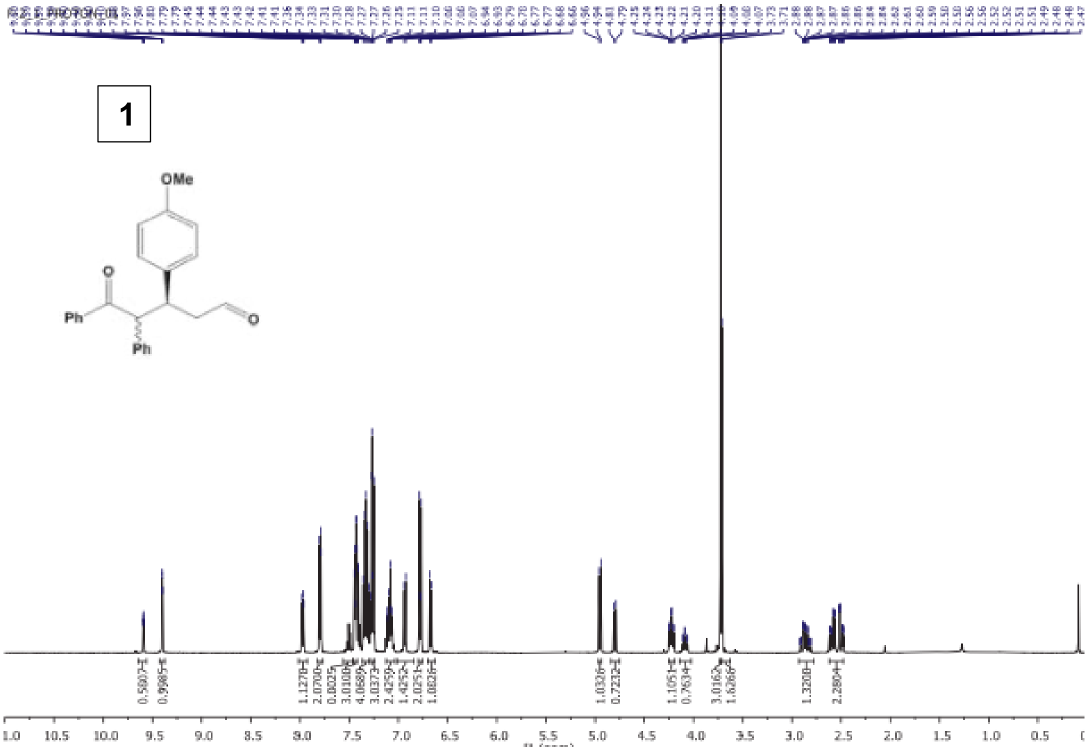

1

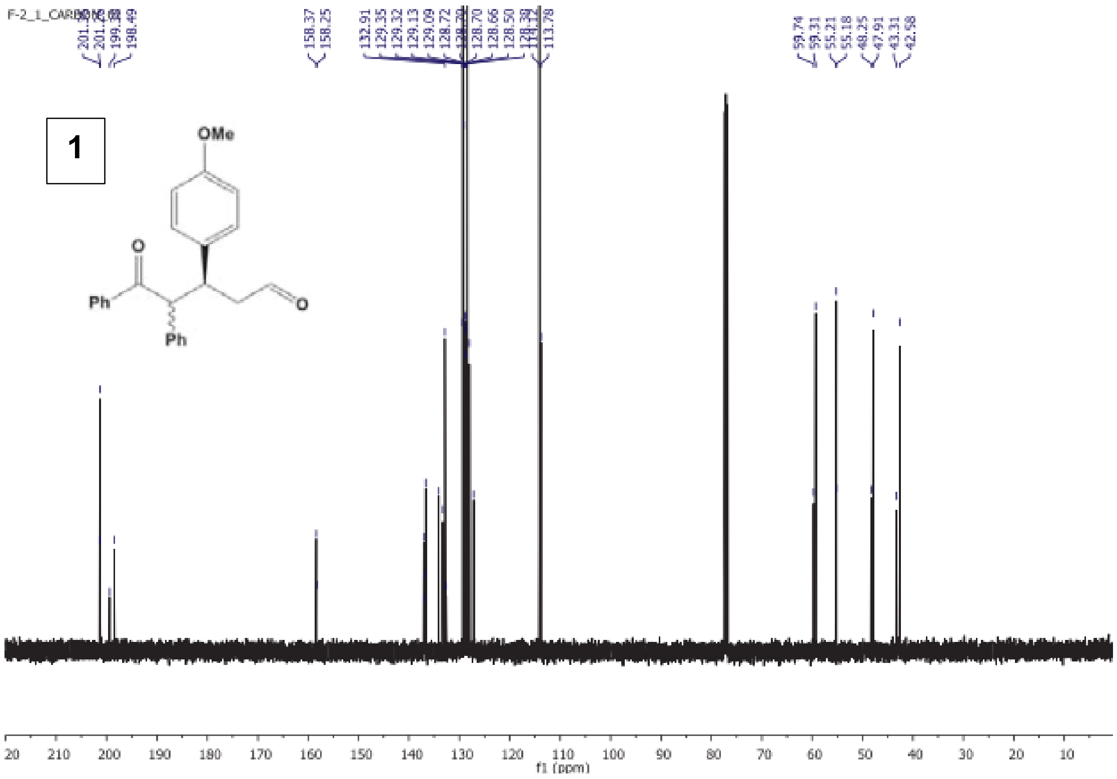

2

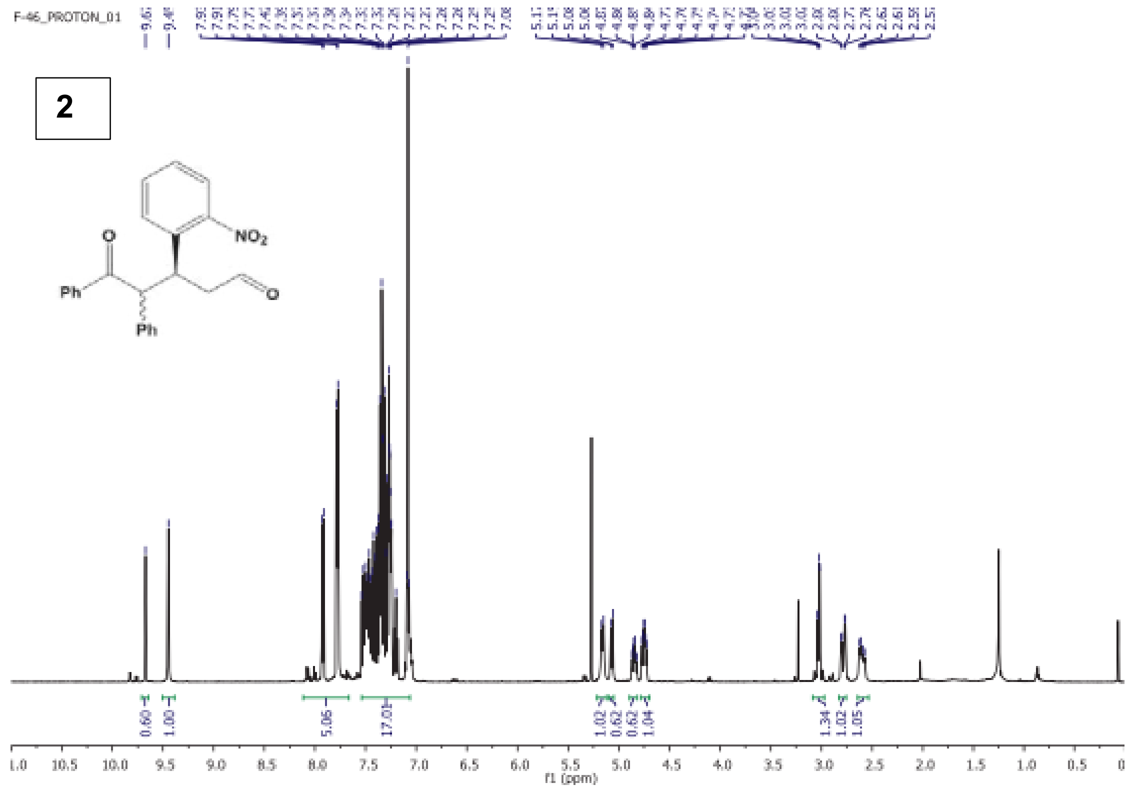

F-46_PROTON_01
NO2
O
Ph
Ph
O
f1 (ppm)

2

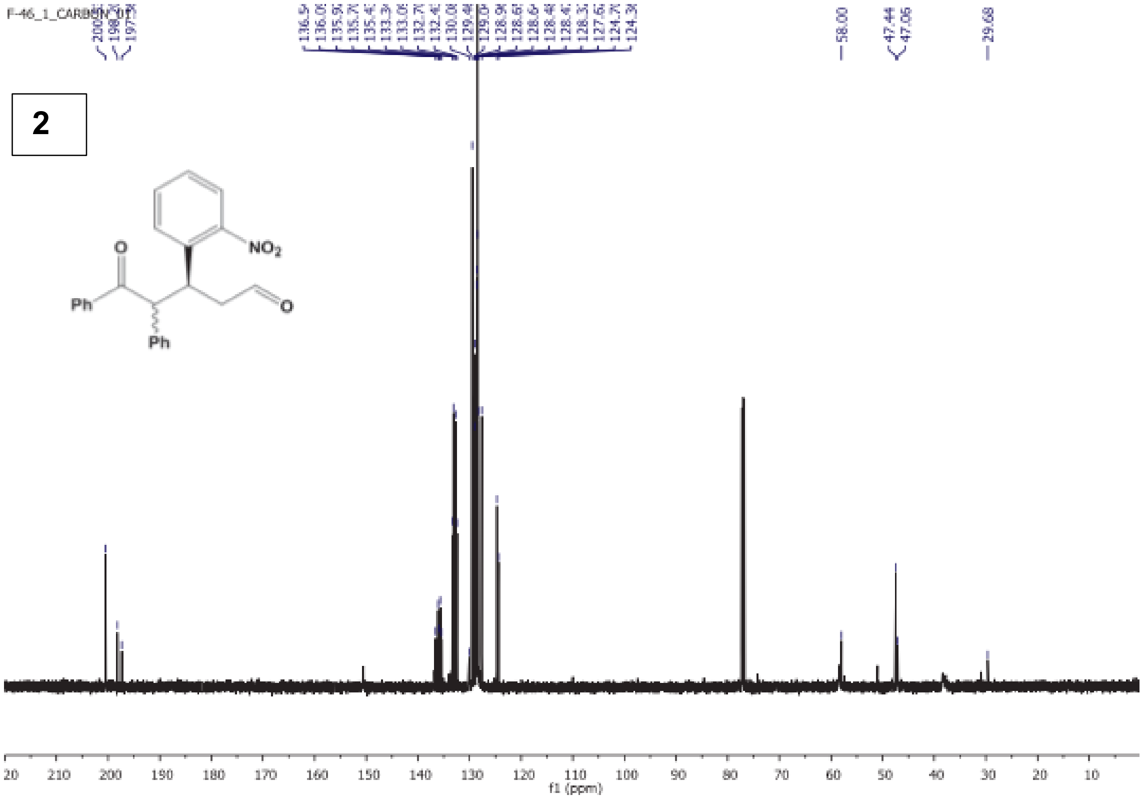

F-46_1_CARBON_01
58.00
47.44
47.06
29.68
NO2
O
Ph
Ph
O
f1 (ppm)

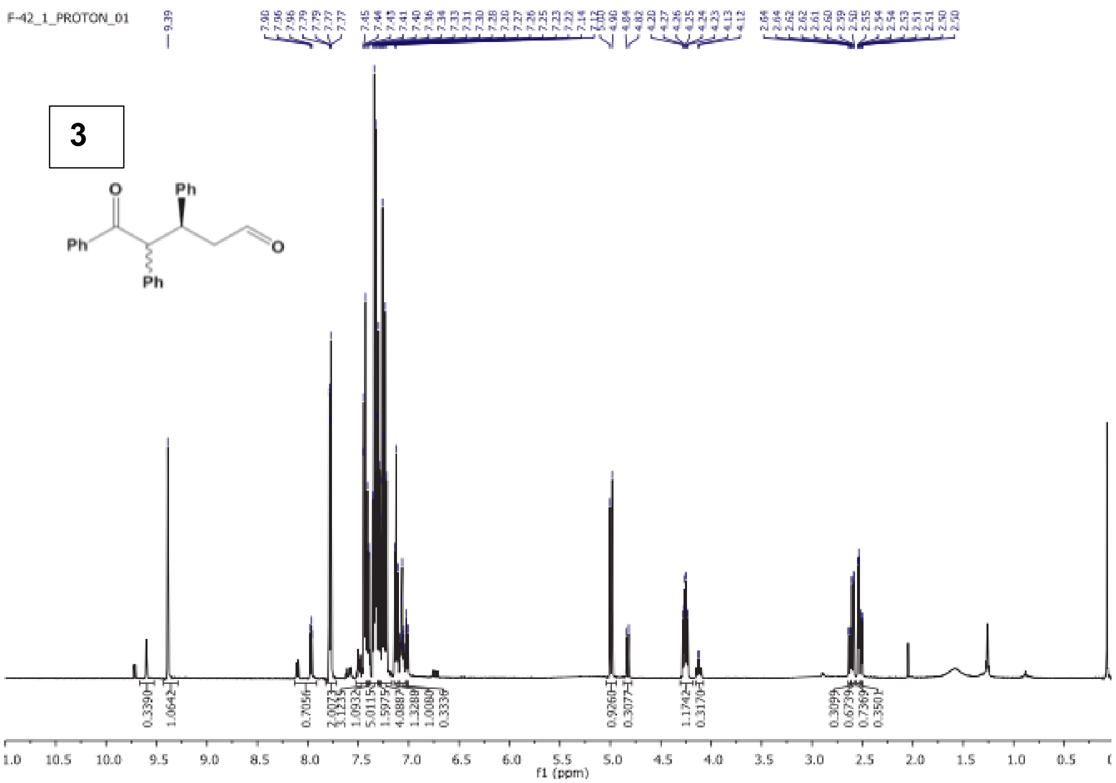
F-42_1_PROTON_01
3
Ph
O
f1 (ppm)

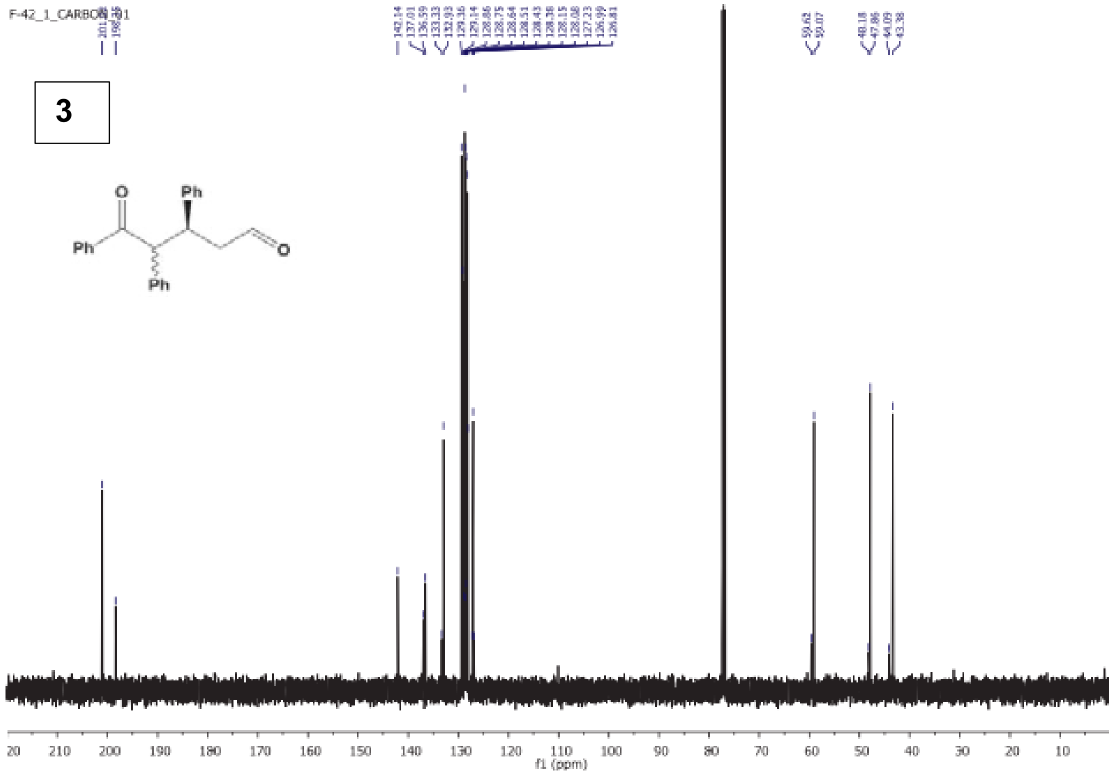
F-42_1_CARBON_01
3
Ph
O
f1 (ppm)

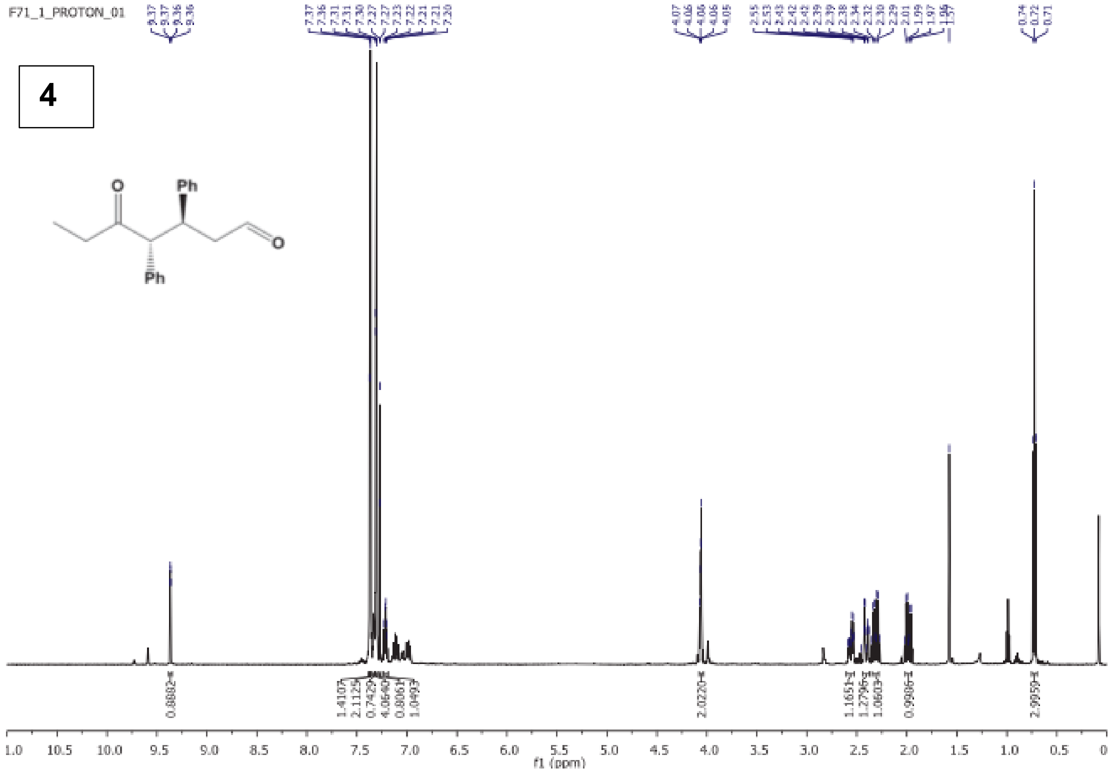
F71_1_PROTON_01
4
O
Ph
Ph
O
f1 (ppm)

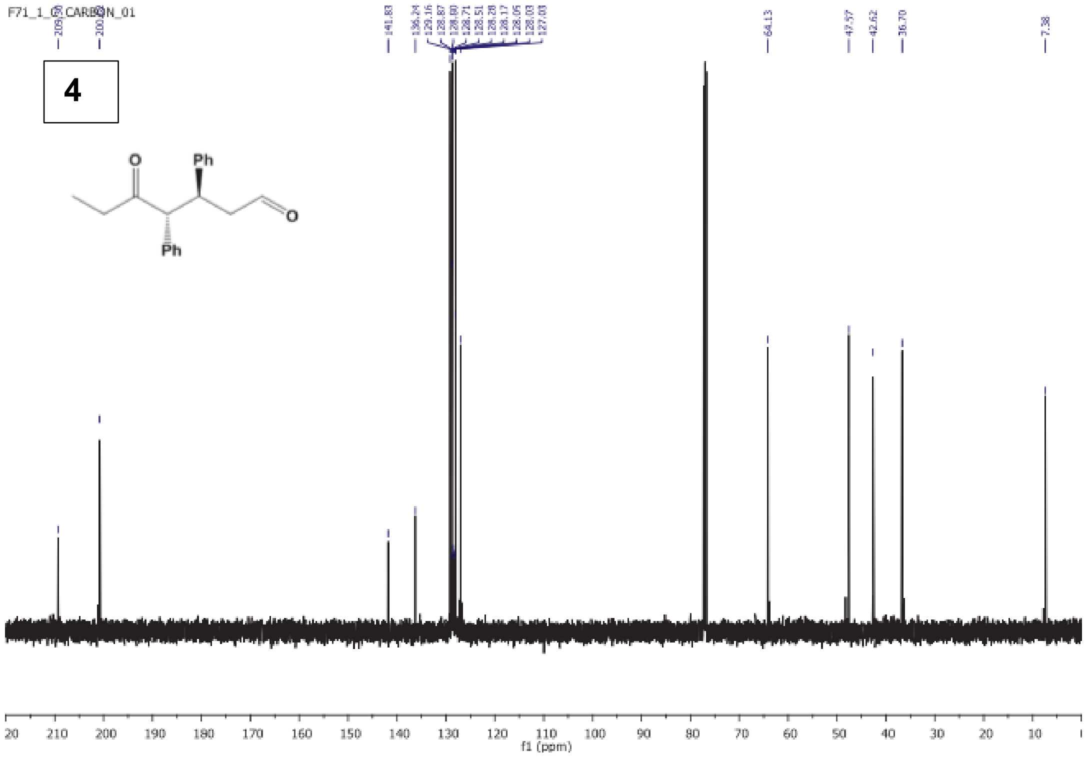
4
O
Ph
Ph
O
f1 (ppm)

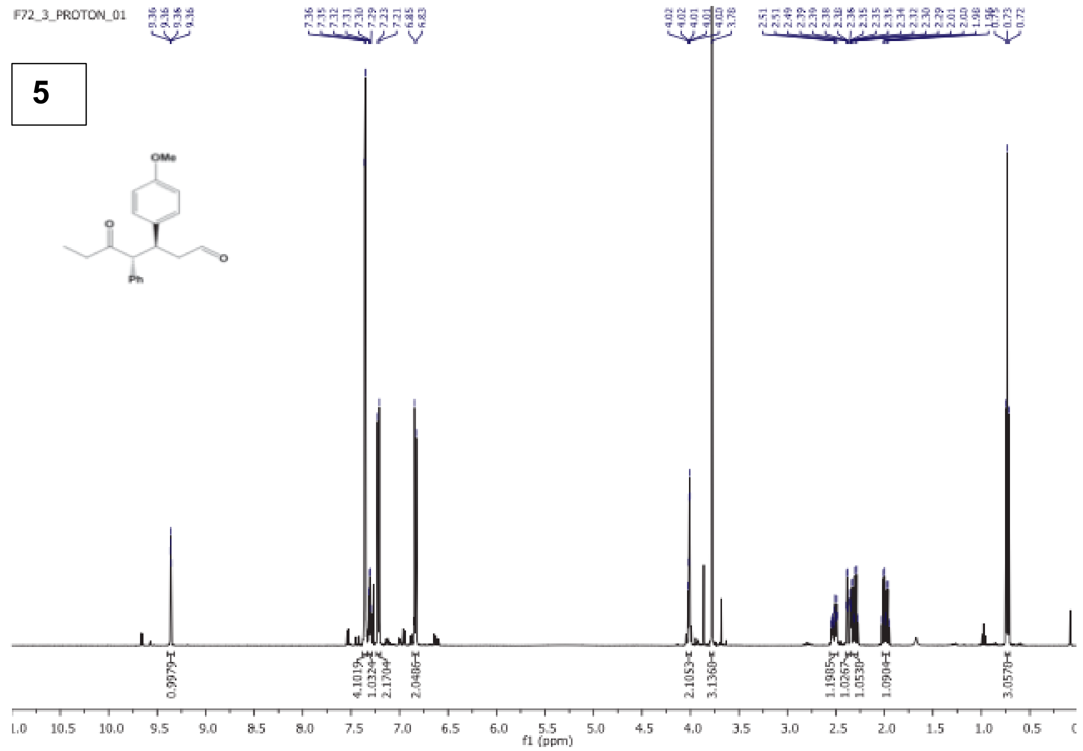
F72_3_PROTON_01
5
OMe
Ph
f1 (ppm)

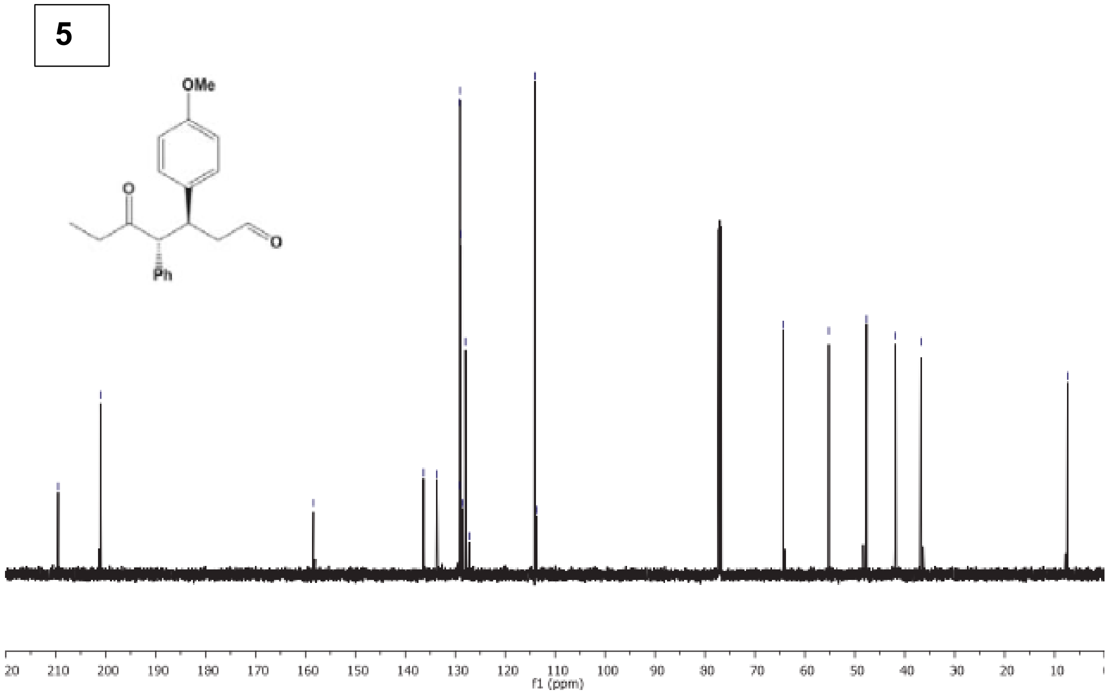
5
OMe
Ph
f1 (ppm)

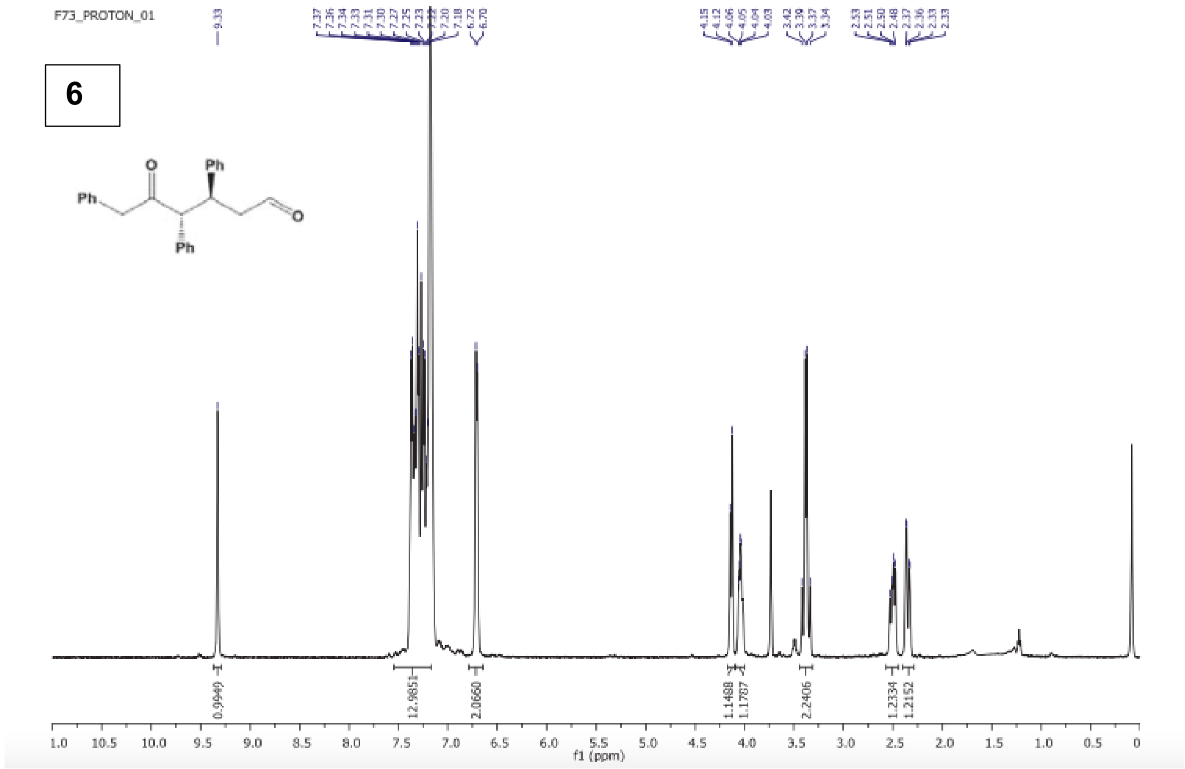
F73_PROTON_01
6
f1 (ppm)

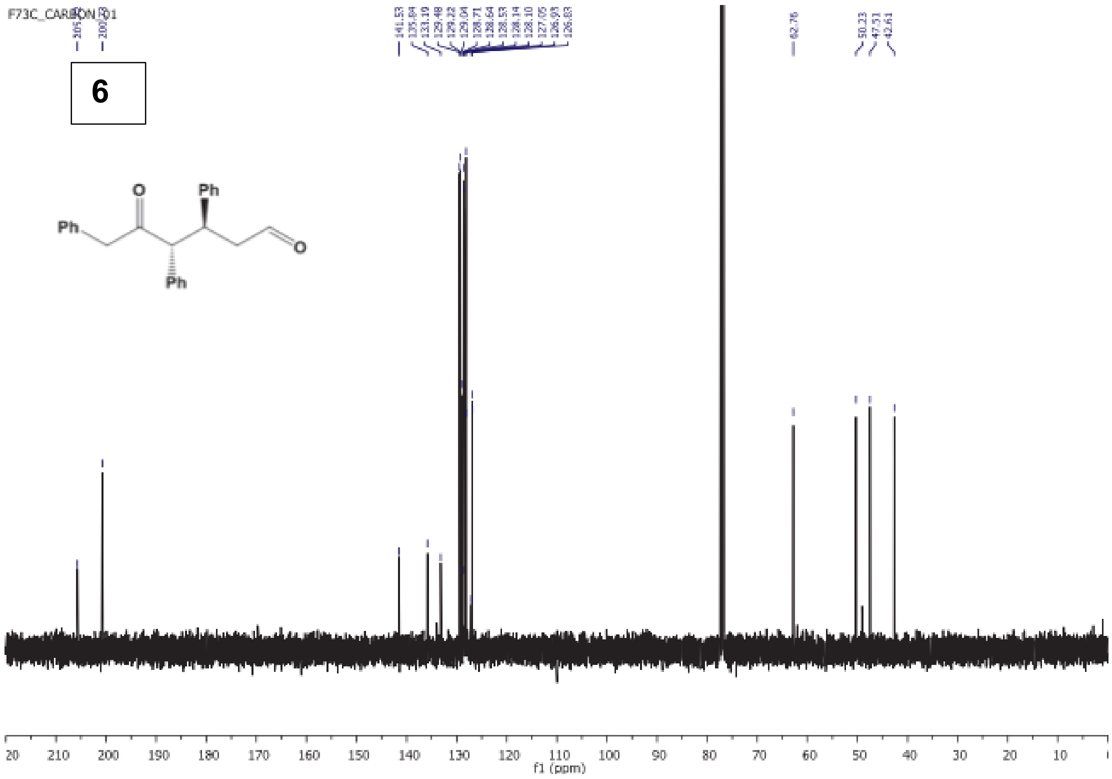
F73C_CARBON_01
6
f1 (ppm)

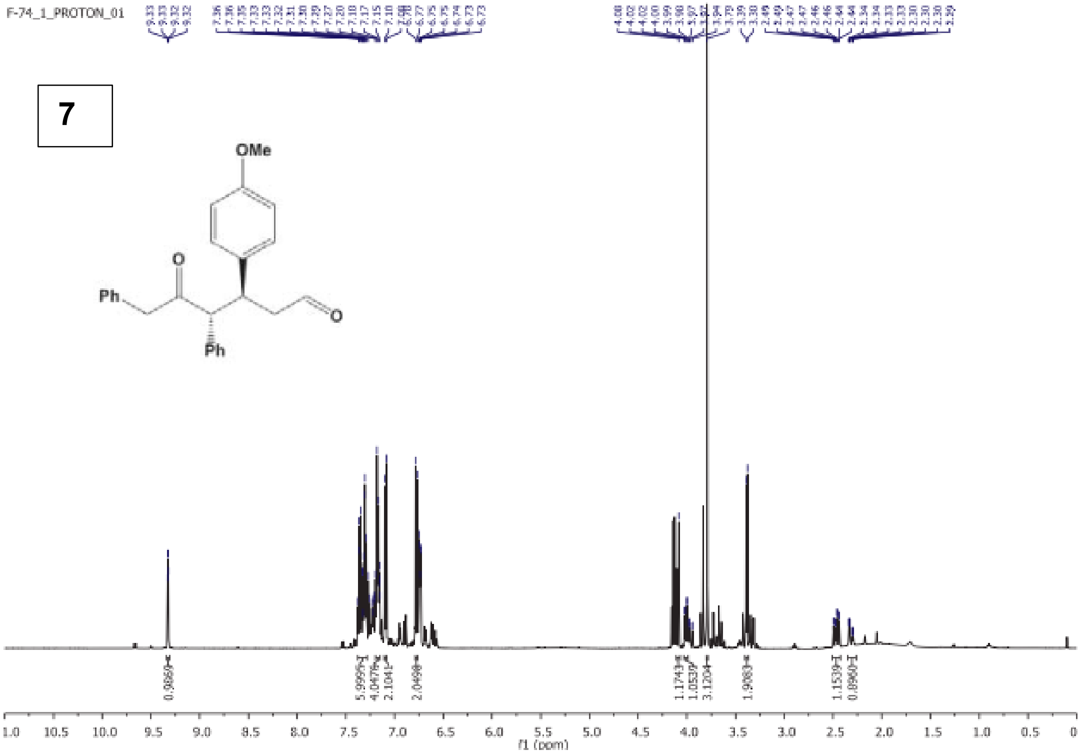
F-74_1_PROTON_01
7
OMe
O
Ph
Ph
O
f1 (ppm)

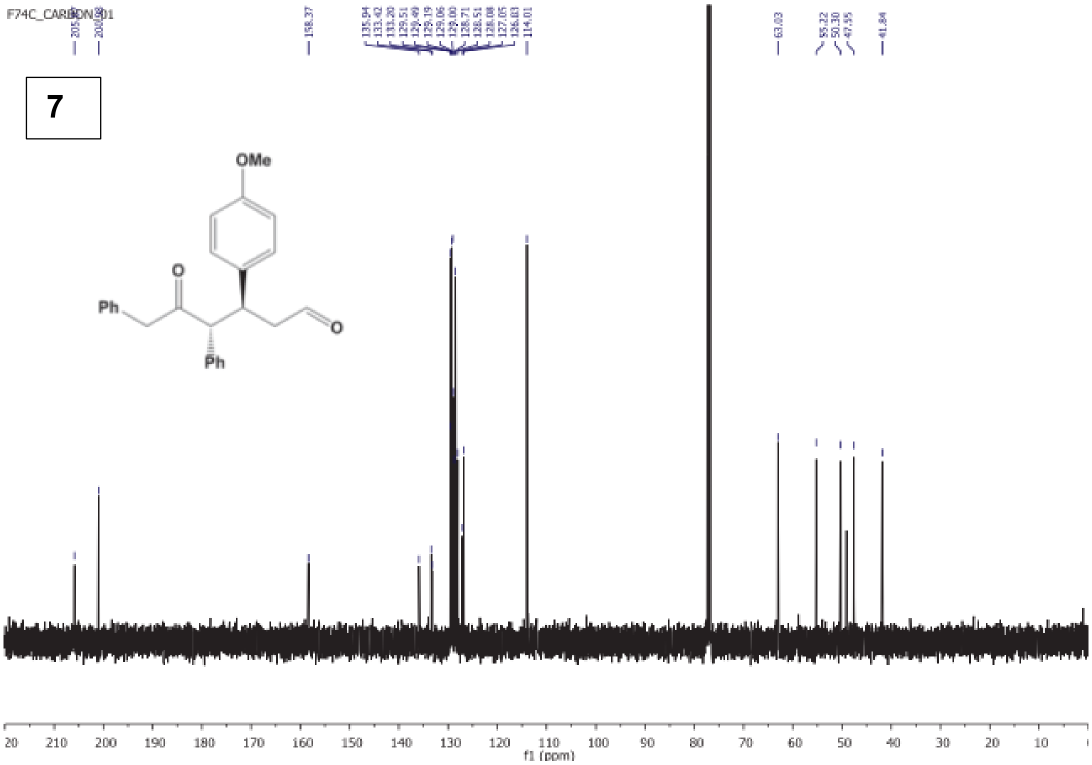
F74C_CARBON_01
7
OMe
O
Ph
Ph
O
f1 (ppm)

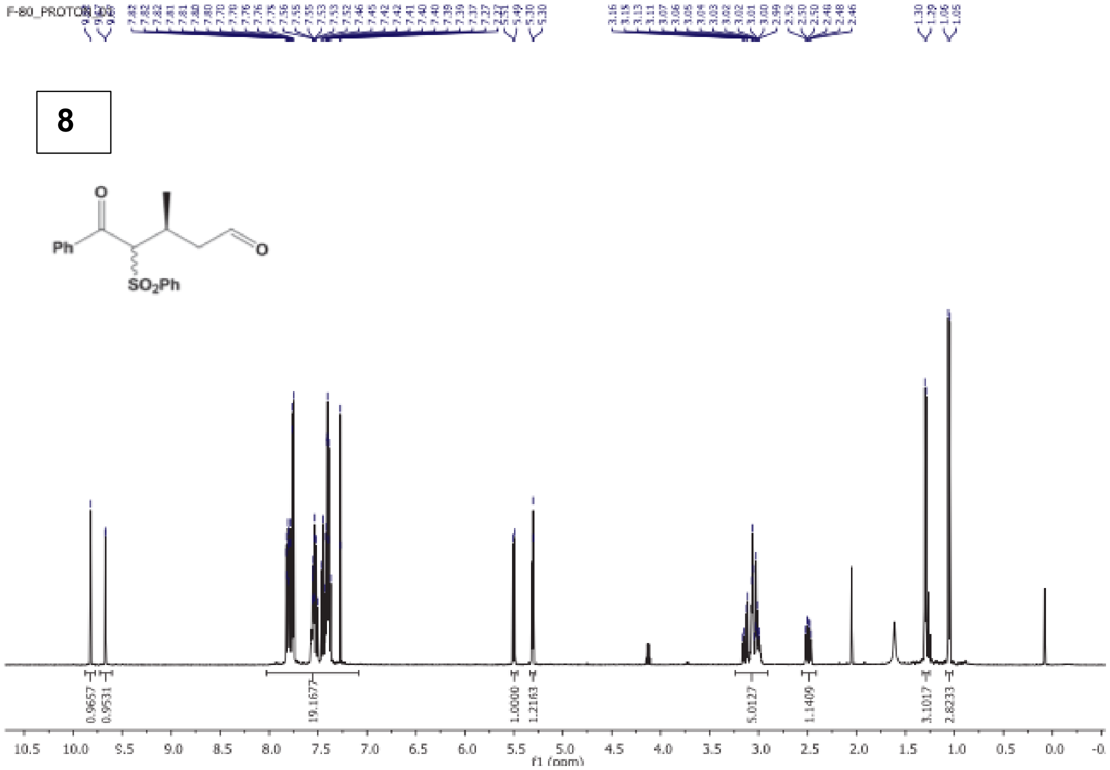
8
Ph
SO2Ph
f1 (ppm)

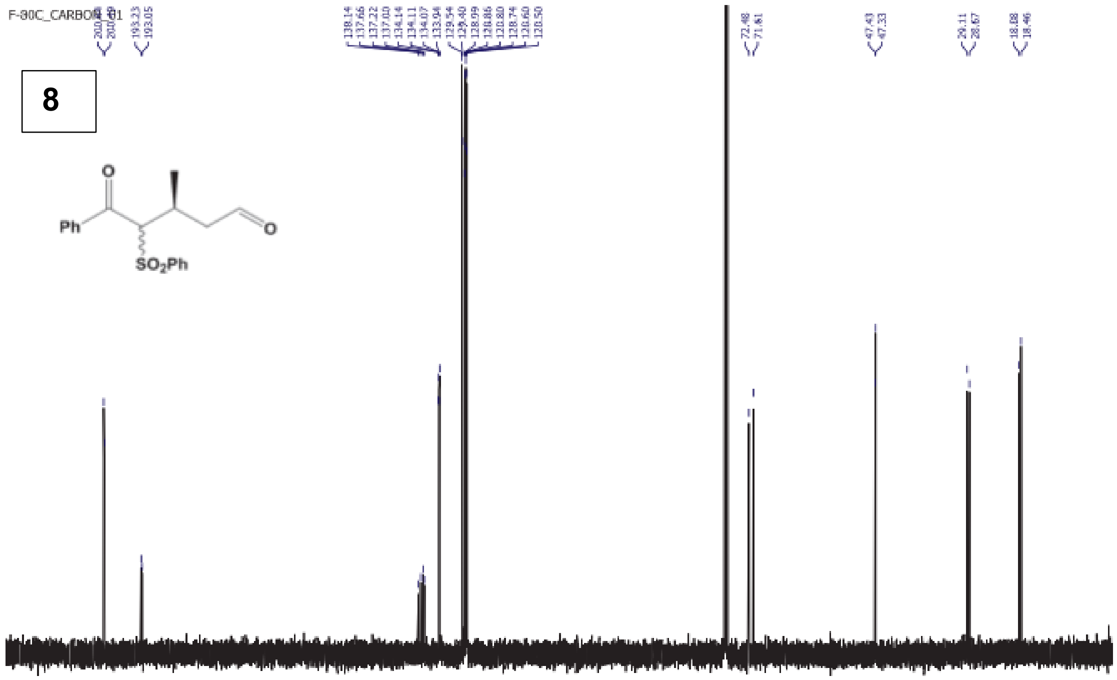
8
Ph
SO2Ph

9

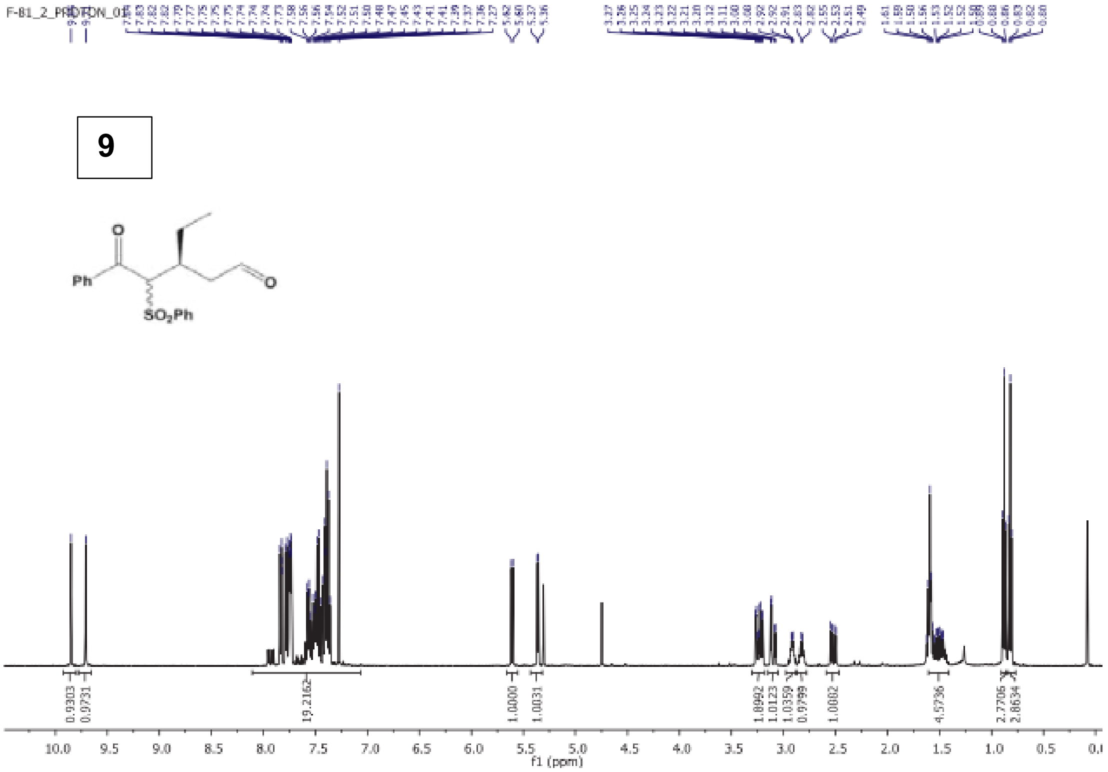

9

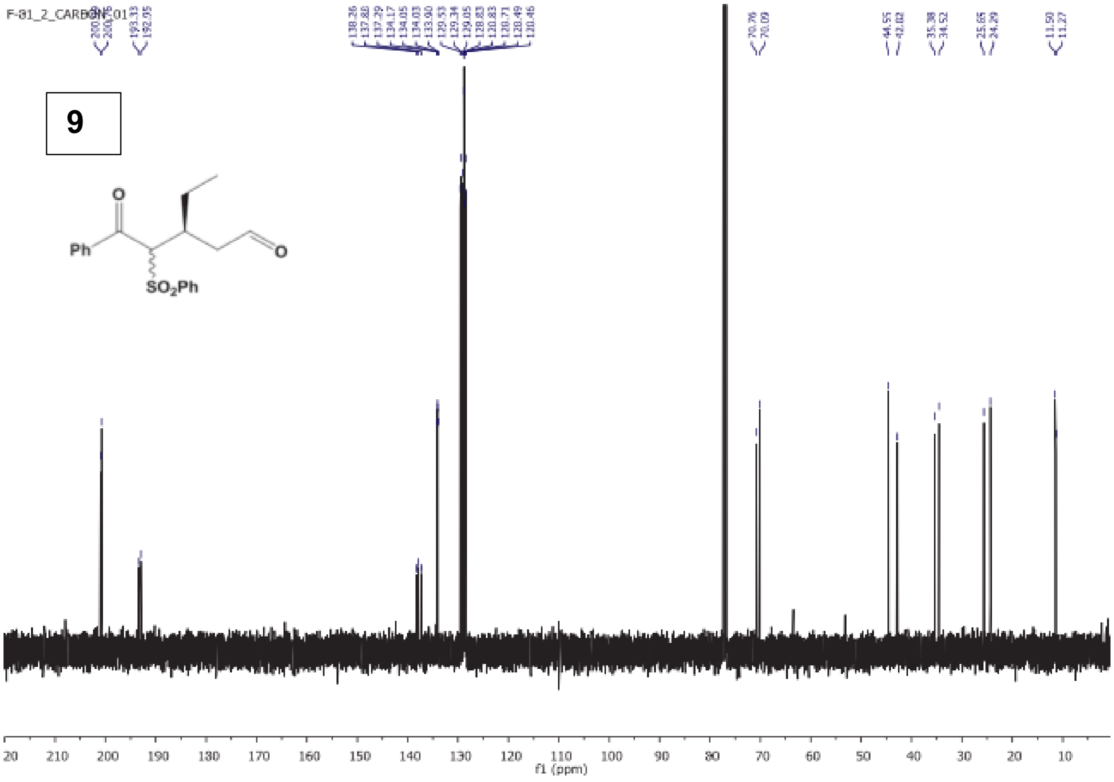

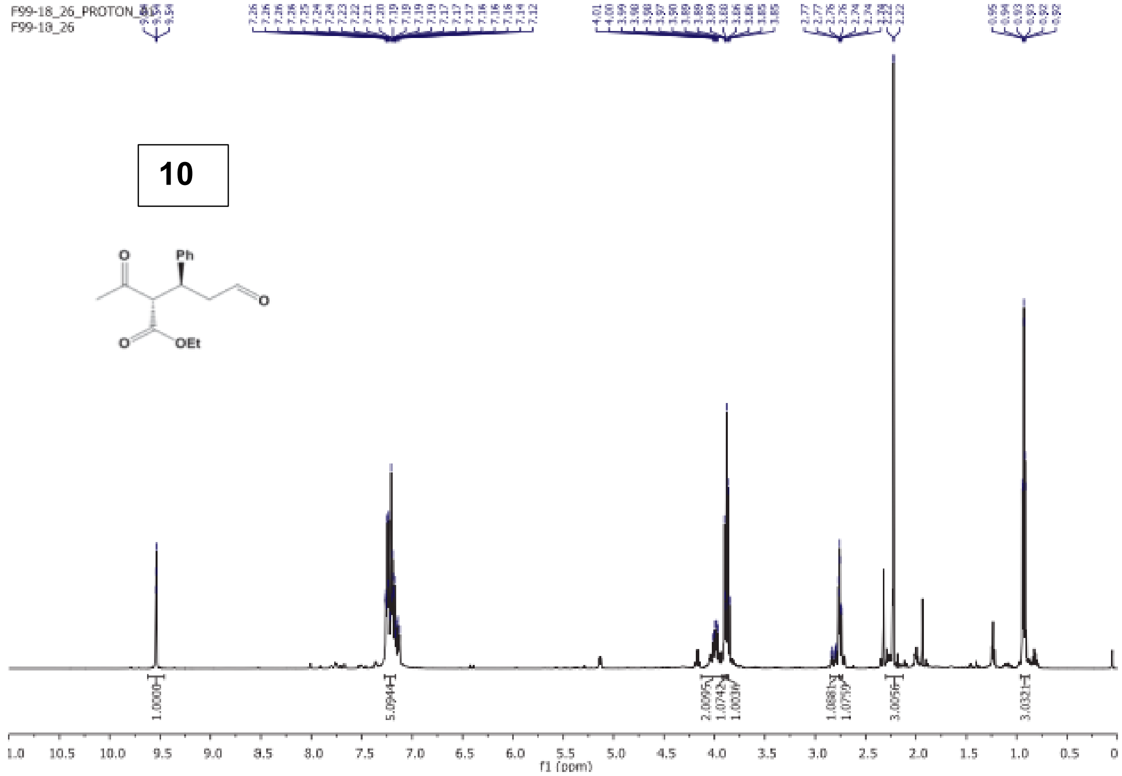
F99-18_26_PROTON_01
F99-18_26
10
Ph
O
OEt
1.0000
5.0944
2.0095
1.0742
1.0030
1.0881
1.0759
3.0056
3.0321
f1 (ppm)

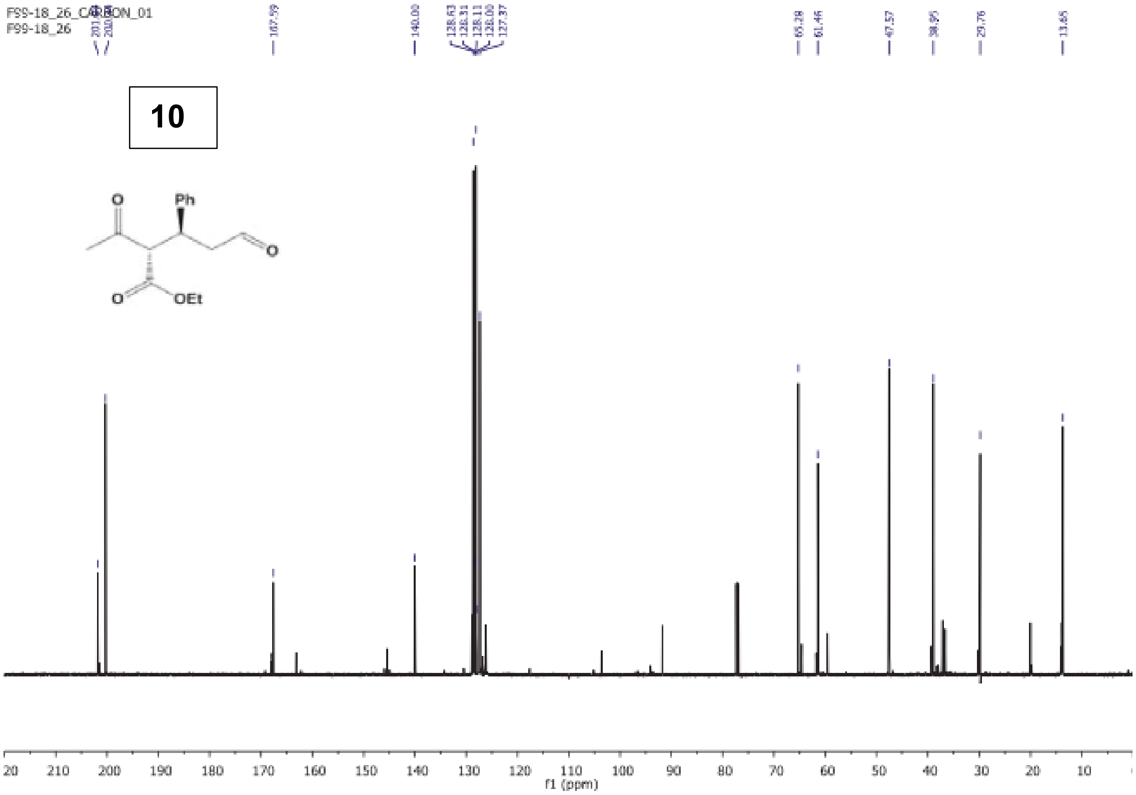
F99-18_26_CARBON_01
F99-18_26
167.59
140.00
128.63
128.31
128.11
128.00
127.37
65.28
61.46
47.57
38.95
29.76
13.65
10
Ph
O
OEt
f1 (ppm)

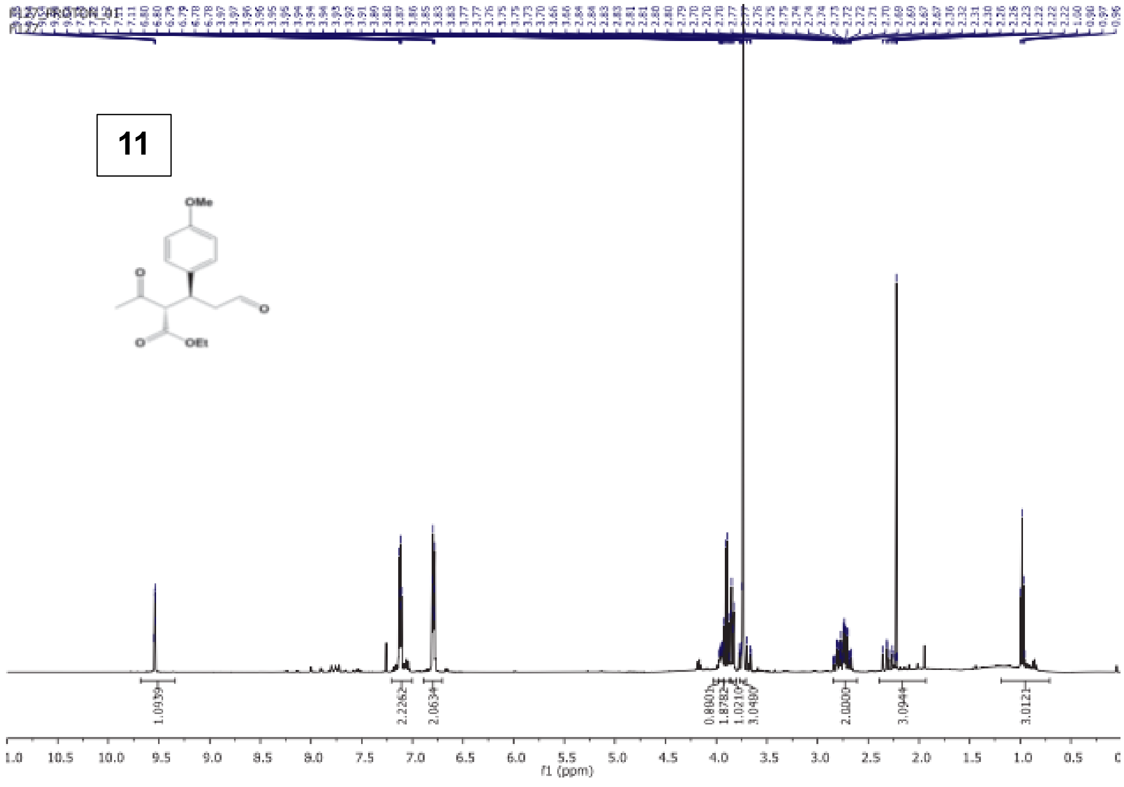
11
OMe
OEt
f1 (ppm)

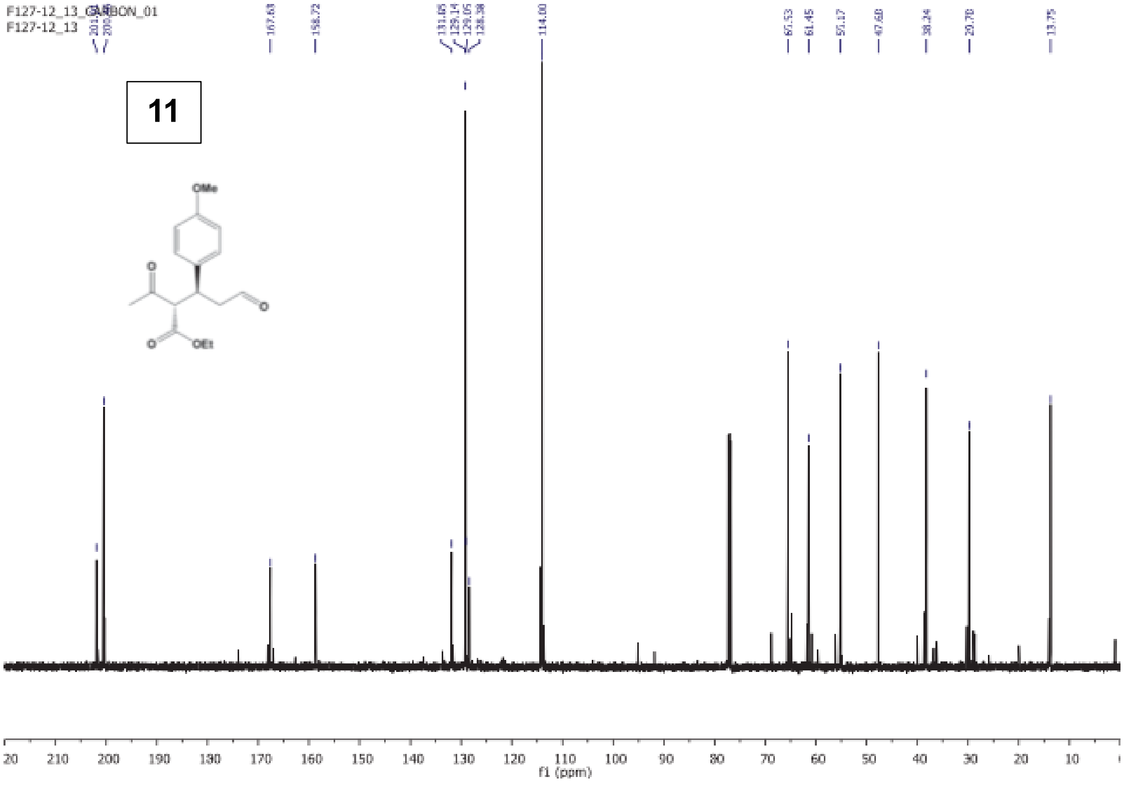
F127-12_13
11
OMe
OEt
f1 (ppm)

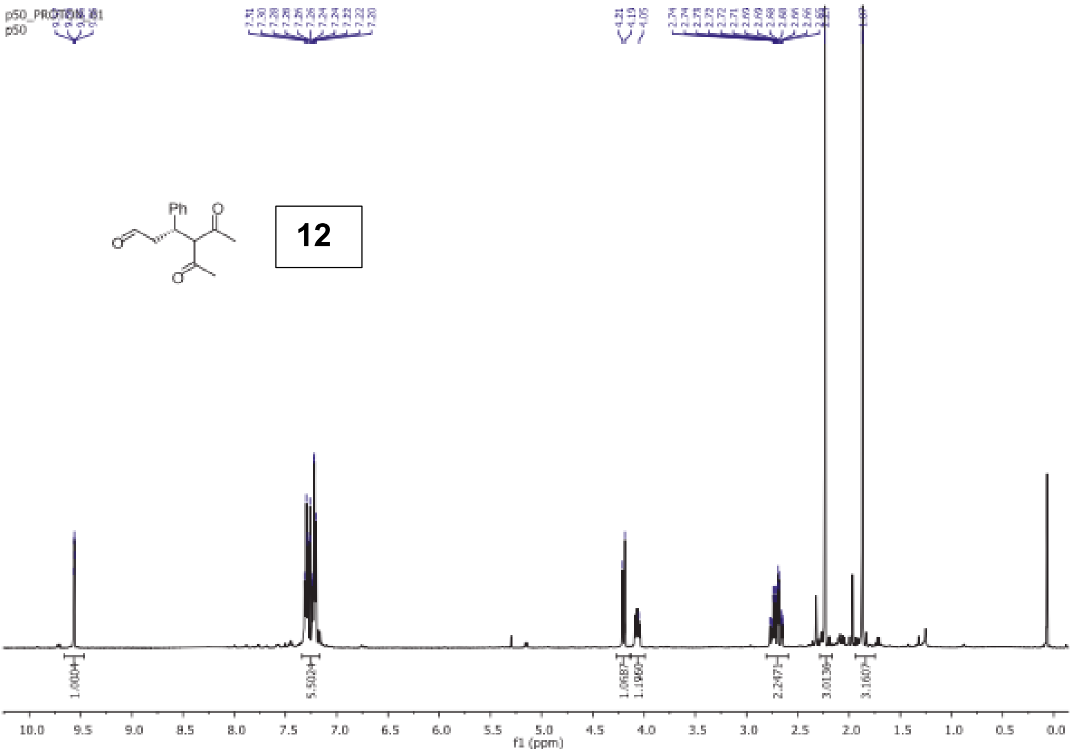
12
f1 (ppm)

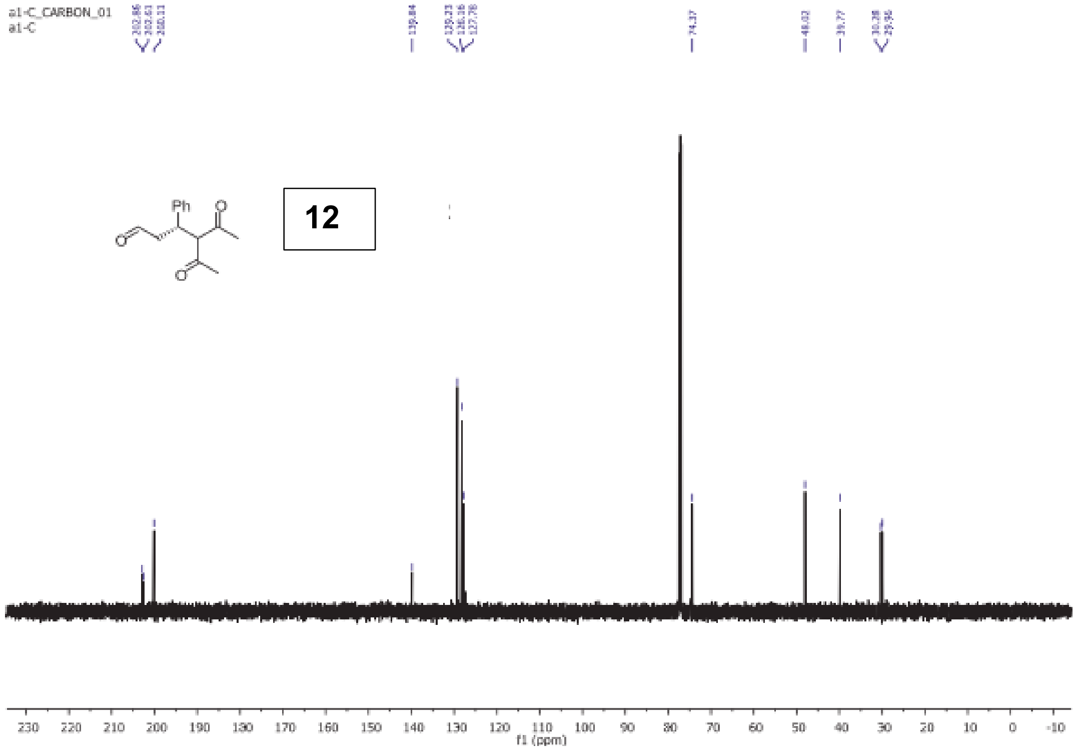
12
f1 (ppm)

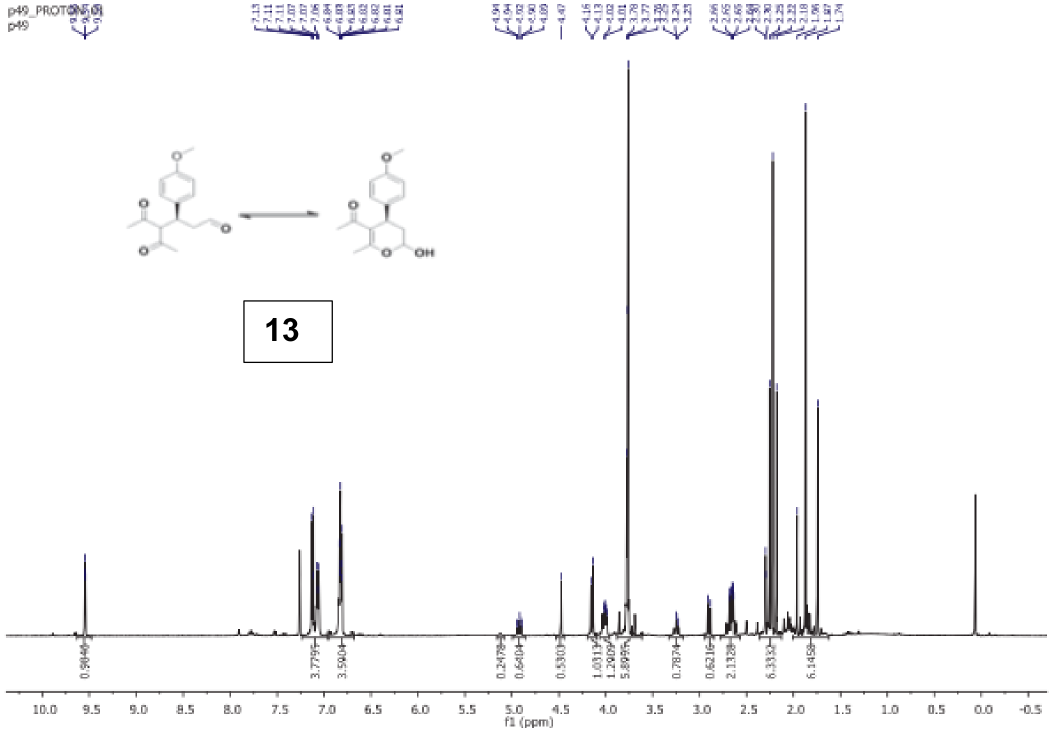
p49
13
f1 (ppm)

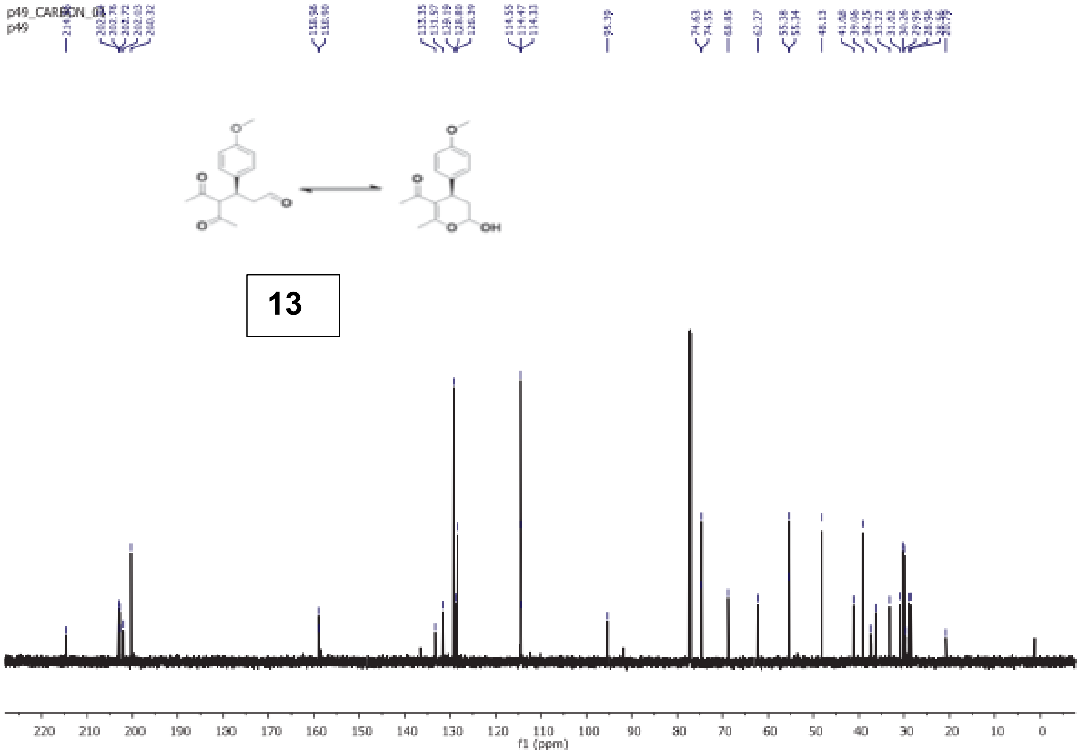
p49
13
f1 (ppm)

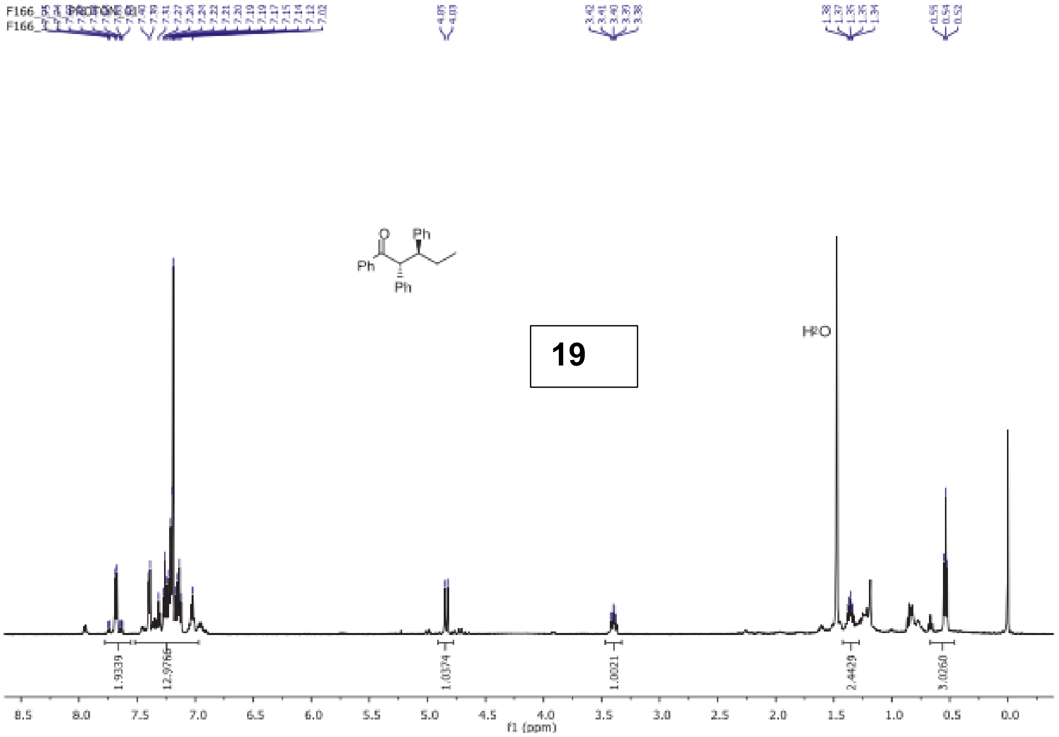
Ph
O
Ph
Ph
19
H2O
4.85
4.83
1.9339
12.9766
1.0374
1.0021
2.4429
3.0260
f1 (ppm)

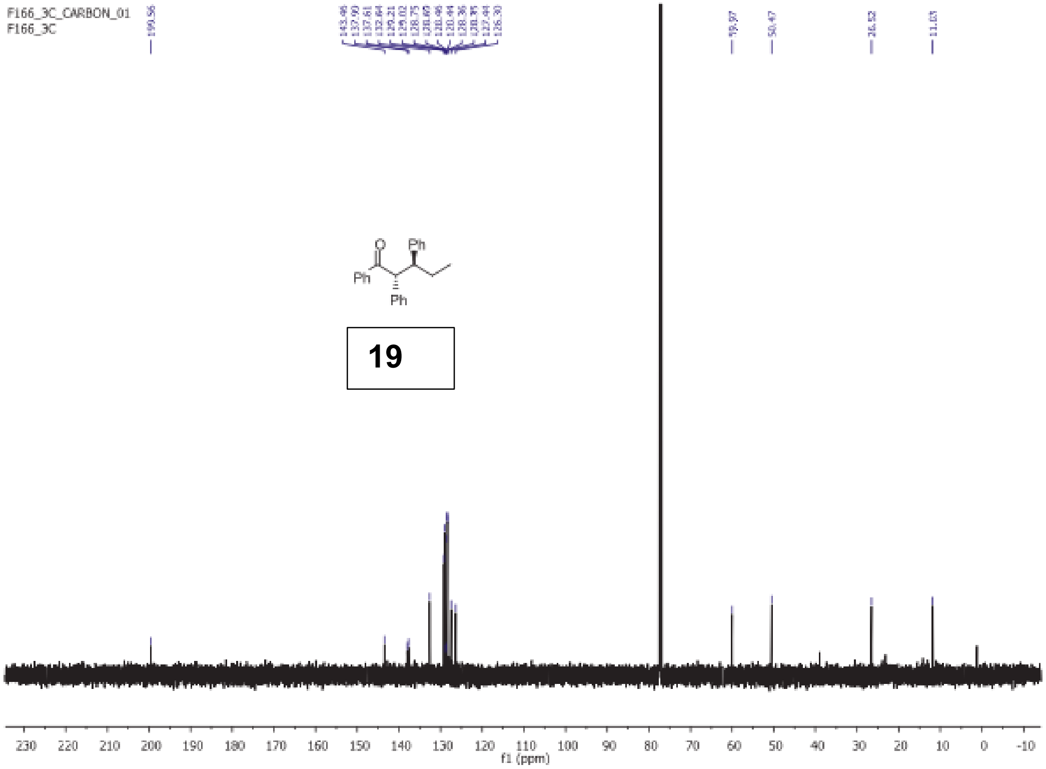
F166_3C_CARBON_01
F166_3C
199.56
59.97
50.47
26.52
11.03
Ph
O
Ph
Ph
19
f1 (ppm)

3. HPLC Chromatograms

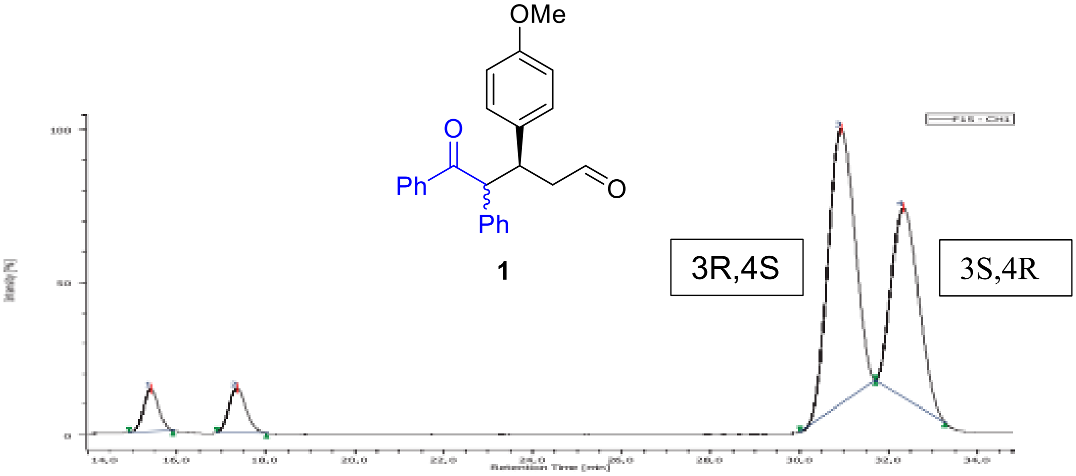

Peak	tR	Area	Height	Area%	Height%	Symmetry
1	15,417	146238	6246	4,579	7,700	1,067
2	17,350	175321	6517	5,490	8,034	1,149
3	30,917	1684049	40490	52,730	49,913	1,026
4	32,317	1188118	27868	37,202	34,354	1,201

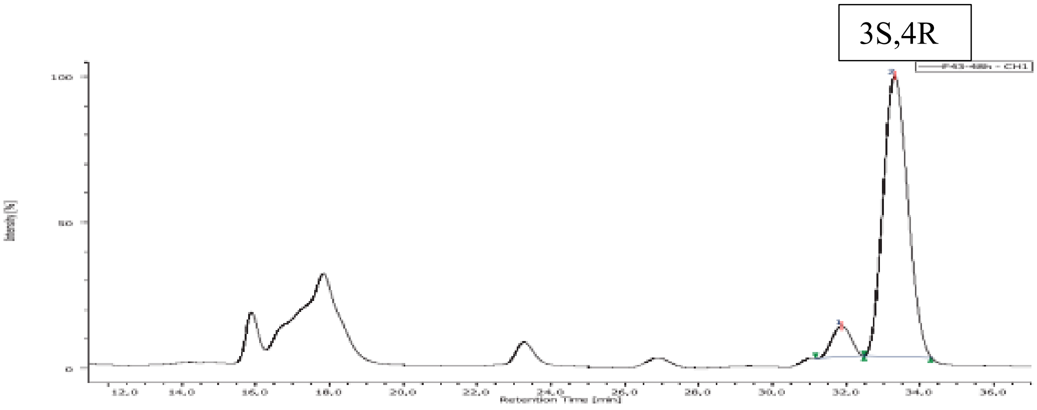

Peak	tR	Area	Height	Area%	Height%	Symmetry
1	31,842	124523	3480	7,843	9,886	0,988
2	33,283	1463092	31722	92,157	90,114	1,115

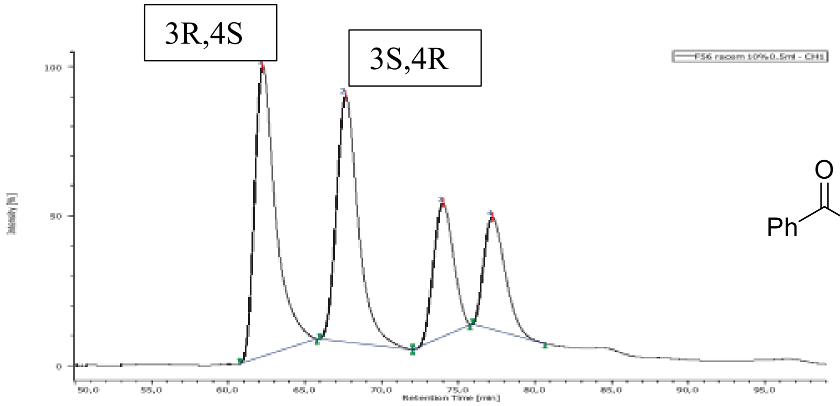

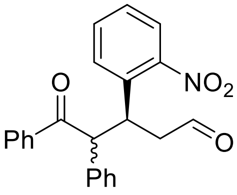

2

tR	Area	Height	Area%	Height%	NTP	Resolution	Symmetry Factor
**62,242**	4369056	45563	37,117	37,161	11380	2,259	1,680
**67,650**	3824967	38671	32,495	31,539	12021	2,569	1,402
**73,967**	1880783	20823	15,978	16,983	14445	1,284	1,101
**77,192**	1696256	17554	14,410	14,317	14381	N/A	1,411

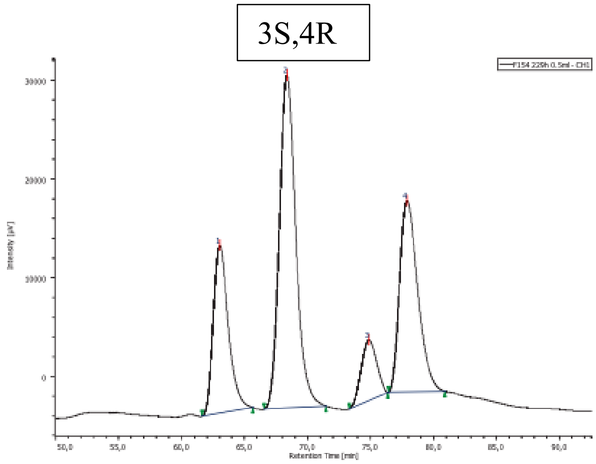

Peak Name	tR	Area	Height	Area%	Height%	NTP	Resolution	Symmetry Factor
**1**	63,017	1406628	16960	20,386	22,312	13837	2,388	1,357
**2**	68,342	3036868	33609	44,013	44,215	13784	2,793	1,209
**3**	74,850	521888	6138	7,564	8,074	16316	1,219	1,010
**4**	77,883	1934588	19307	28,038	25,399	13855	N/A	1,370

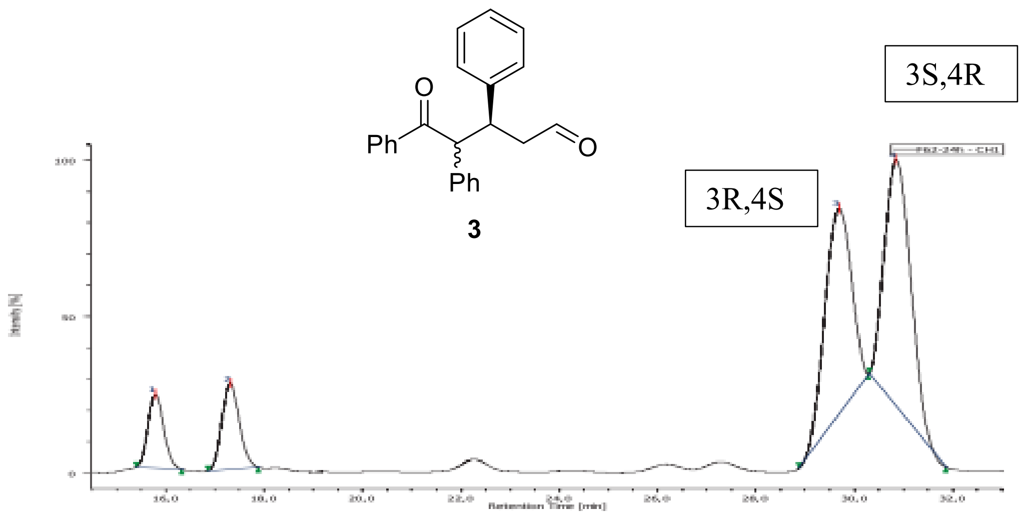

Peak	tR	Area	Height	Area%	Height%	Symmetry
1	15.792	166253	7487	7.951	12.063	1.108
2	17.308	214082	8725	10.238	14.059	1.114
3	29.667	780952	20852	37.348	33.600	0.938
4	30.825	929724	24996	44.463	40.277	1.165

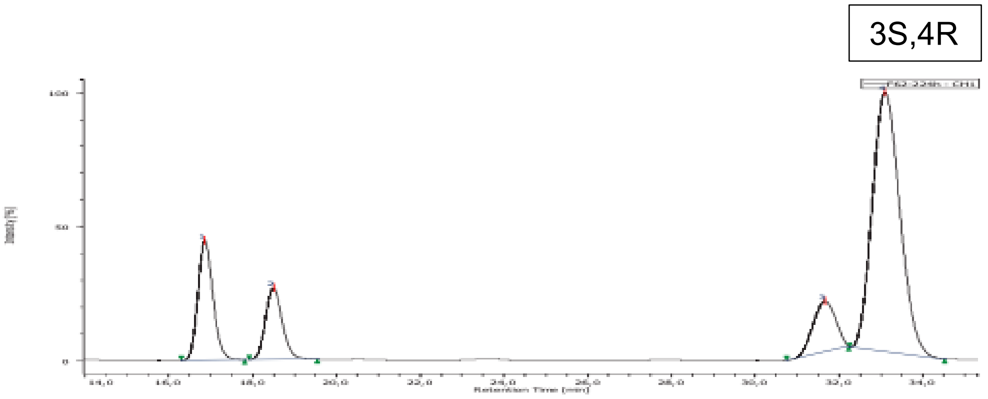

Peak	tR	Area	Height	Area%	Height%	Symmetry
1	16,858	1155221	45856	16,031	23,858	1,159
2	18,492	769135	27452	10,673	14,283	1,151
3	31,642	734199	19398	10,188	10,092	0,935
4	33,075	4547663	99496	63,107	51,766	1,148

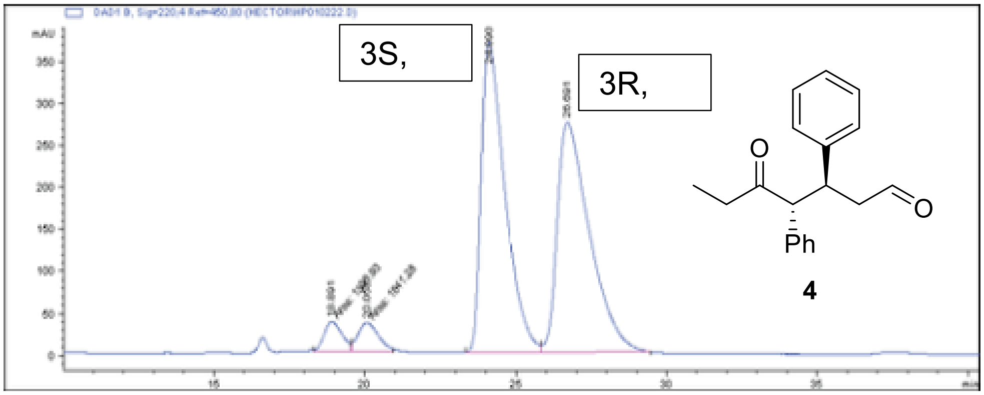

Peak	Time	Area	Height	Width	Area%	Symmetry
1	18.891	1506.9	36	0.6969	3.433	0.742
2	20.058	1641.3	34.8	0.7863	3.739	0.748
3	24.09	20169.2	370.4	0.7728	45.942	0.431
4	26.691	20583.7	274.8	1.0052	46.886	0.428

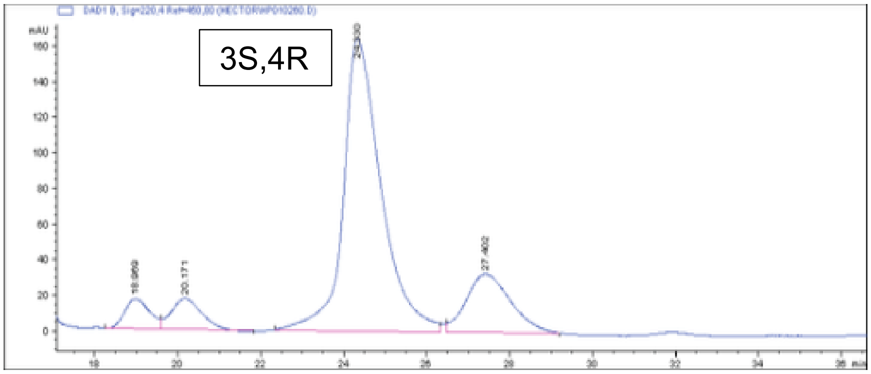

Peak	Time	Area	Height	Width	Area%	Symmetry
1	18.969	717.3	16.9	0.5124	4.962	0.704
2	20.171	895.1	17.5	0.6286	6.192	0.804
3	24.33	10388.1	164.2	0.8406	71.853	0.625
4	27.402	2456.8	32.9	0.894	16.994	0.712

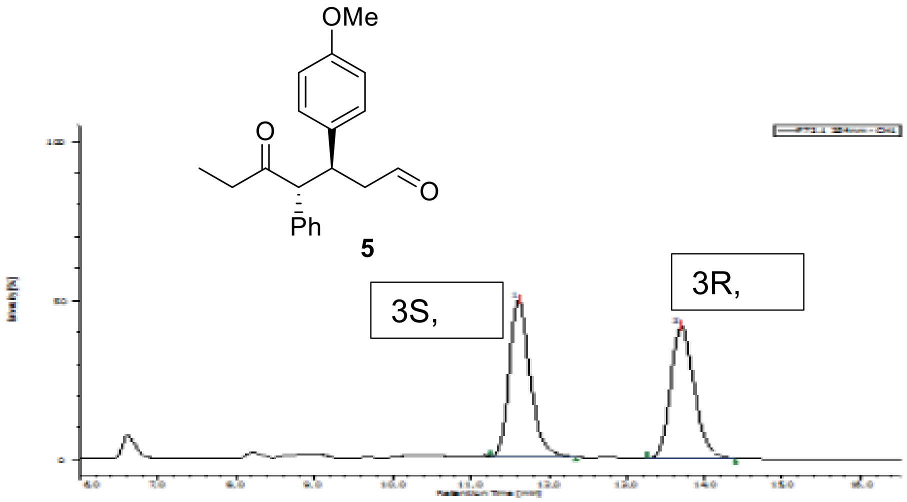

Peak	tR	Area	Height	Area%	Height%	Symmetry
1	11,608	152980	8290	51,211	54,276	1,271
2	13,692	145748	6984	48,789	45,724	1,197

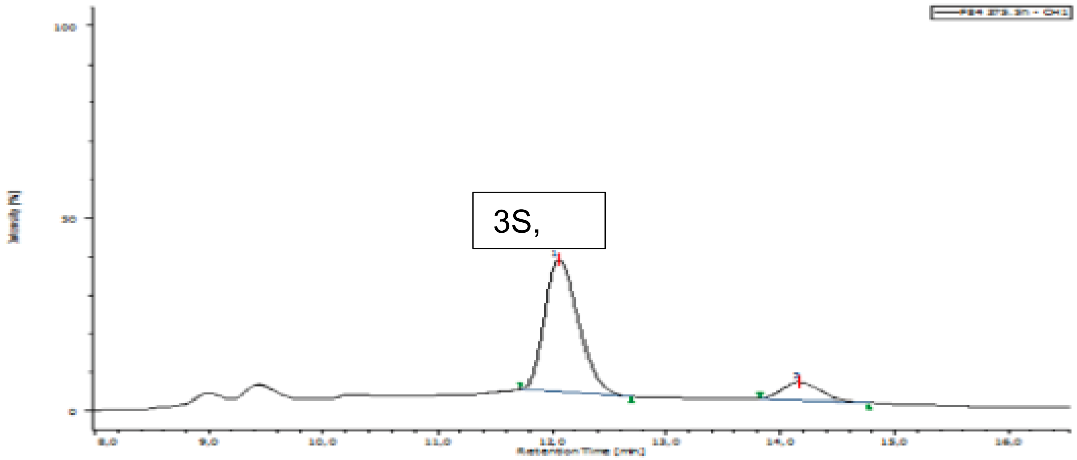

Peak	tR	Area	Height	Area%	Height%	Symmetry
1	12,058	197346	9487	87,020	88,189	1,281
2	14,158	29436	1271	12,980	11,811	1,209

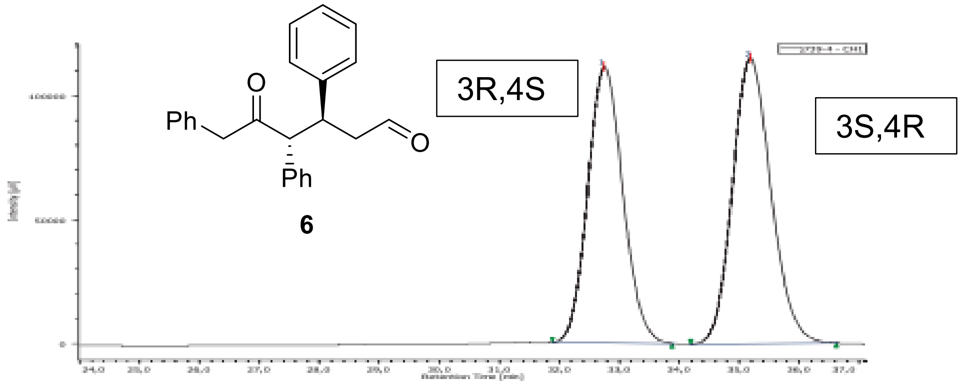

Peak	tR	Area	Height	Area%	Height%	Symmetry
1	32,742	4521136	111366	47,004	49,219	1,098
2	35,167	5097518	114899	52,996	50,781	1,155

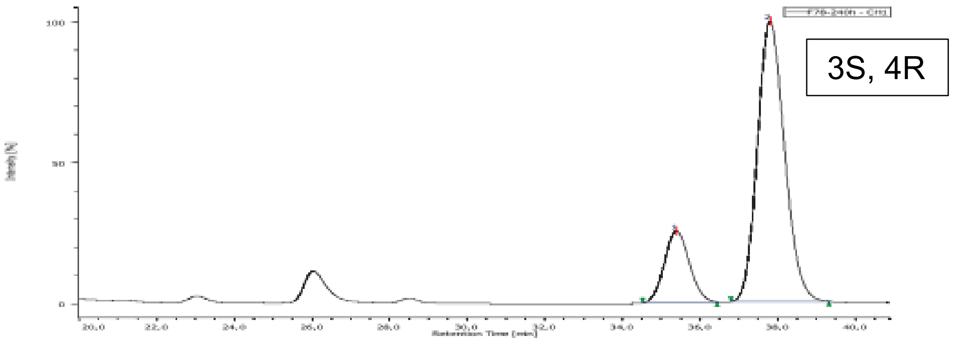

Peak	tR	Area	Height	Area%	Height%	Symmetry
1	35.375	946881	21173	18.902	20.293	1.065
2	37.783	4062604	83163	81.098	79.707	1.154

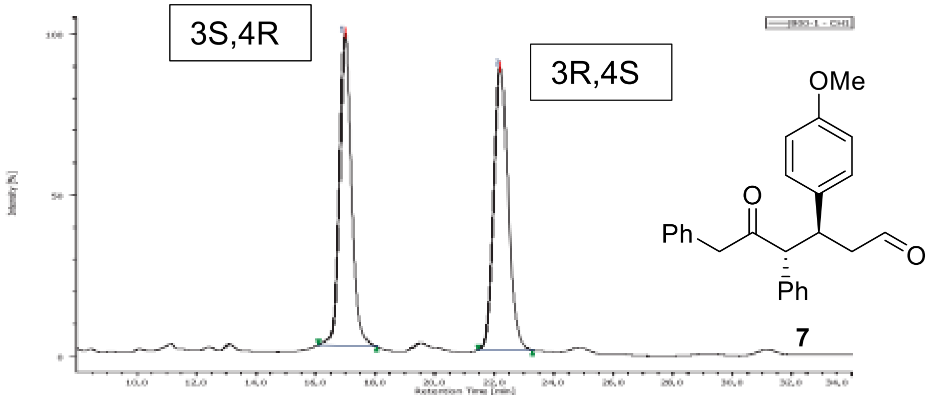

Peak	tR	Area	Height	Area%	Height%	Symmetry
1	16,975	2093289	74473	47,711	52,444	1,176
2	22,192	2294169	67531	52,289	47,556	1,123

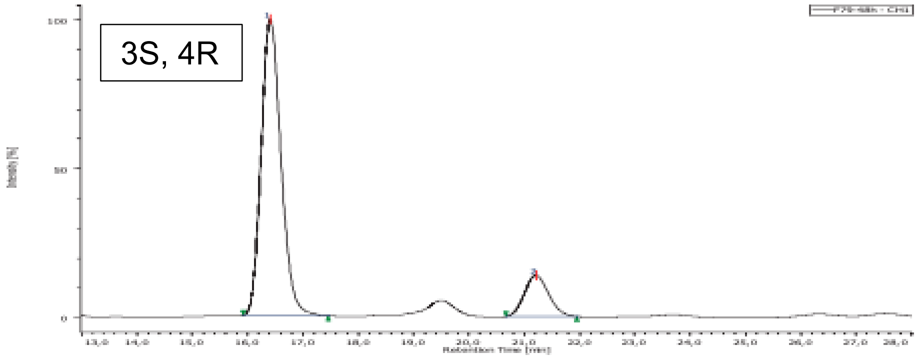

Peak	tR	Area	Height	Area%	Height%	Symmetry
1	16.400	13709580	545474	85.320	87.722	1.196
2	21.200	2358772	76344	14.680	12.278	1.123

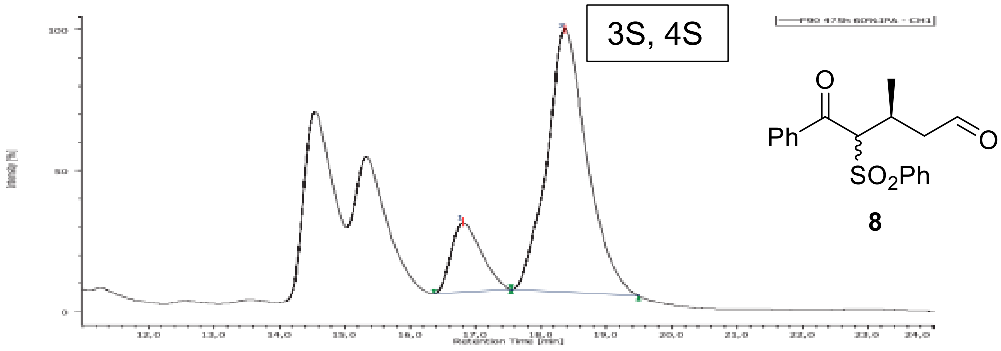

Peak	tR	Area	Height	Area%	Height%	Symmetry
1	16,800	532356	16749	16,719	21,022	1,381
2	18,358	2651698	62927	83,281	78,978	1,144

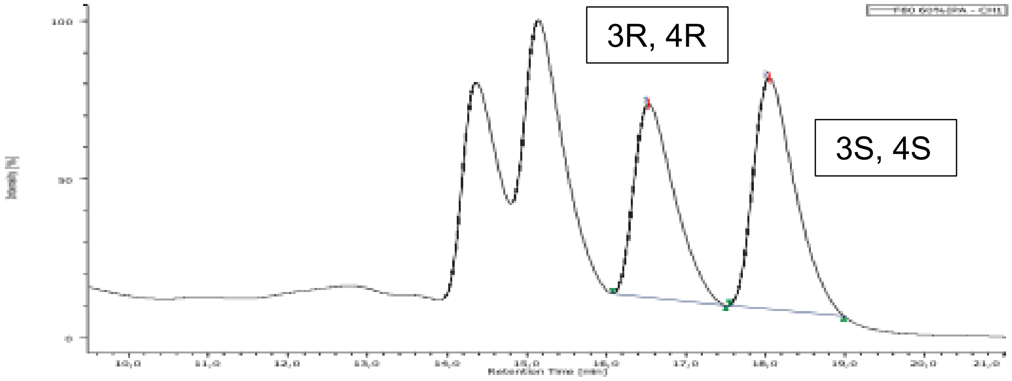

Peak	tR	Area	Height	Area%	Height%	Symmetry
1	16,525	618464	17489	45,605	45,771	1,565
2	18,033	737658	20720	54,395	54,229	1,444

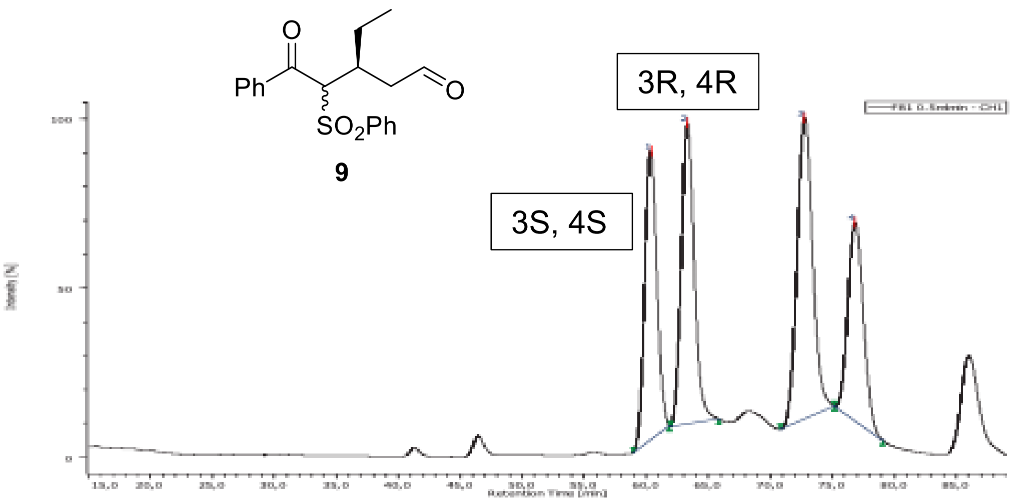

Peak	tR	Area	Height	Area%	Height%	Symmetry
1	60,300	11857175	172685	23,166	26,510	1,081
2	63,267	13162907	180153	25,717	27,656	1,166
3	72,717	15761890	179770	30,795	27,598	1,102
4	76,783	10400826	118789	20,321	18,236	1,109

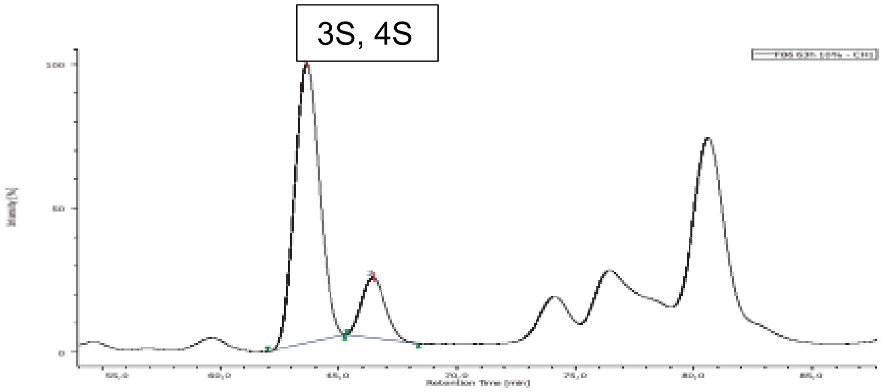

Peak	tR	Area	Height	Area%	Height%	Symmetry
1	63.625	1114737	15372	82.626	82.227	1.074
2	66.408	234402	3323	17.374	17.773	1.150

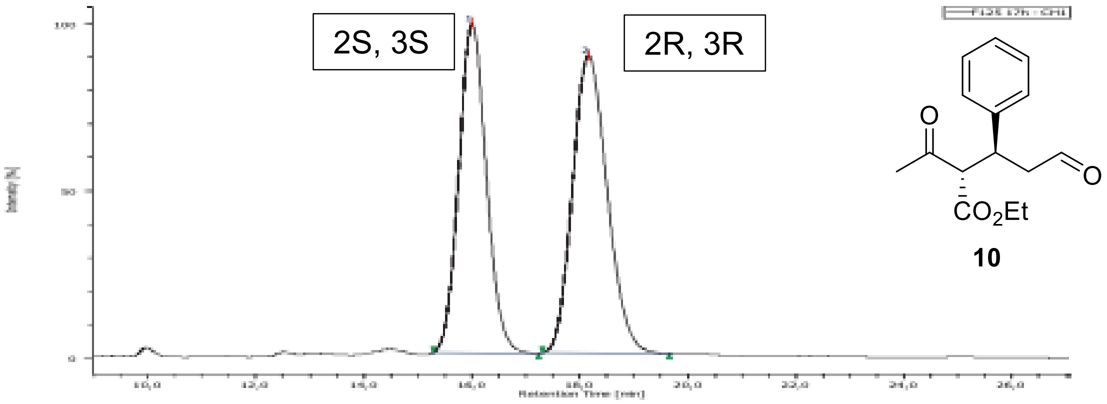

Peak	tR	Area	Height	Area%	Height%	Symmetry
1	15.992	4578360	129342	46.761	52.521	1.093
2	18.150	5212637	116924	53.239	47.479	1.160

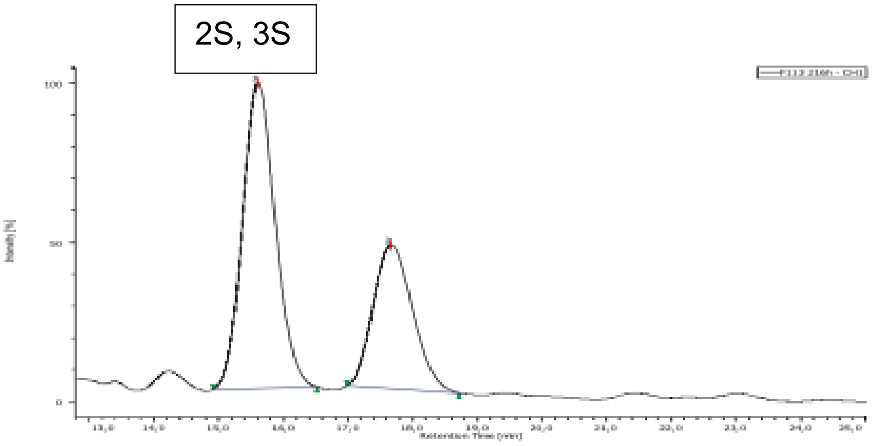

Peak	tR	Area	Height	Area%	Height%	Symmetry
1	15,608	812803	23073	63,870	68,006	1,105
2	17,658	459789	10855	36,130	31,994	1,179

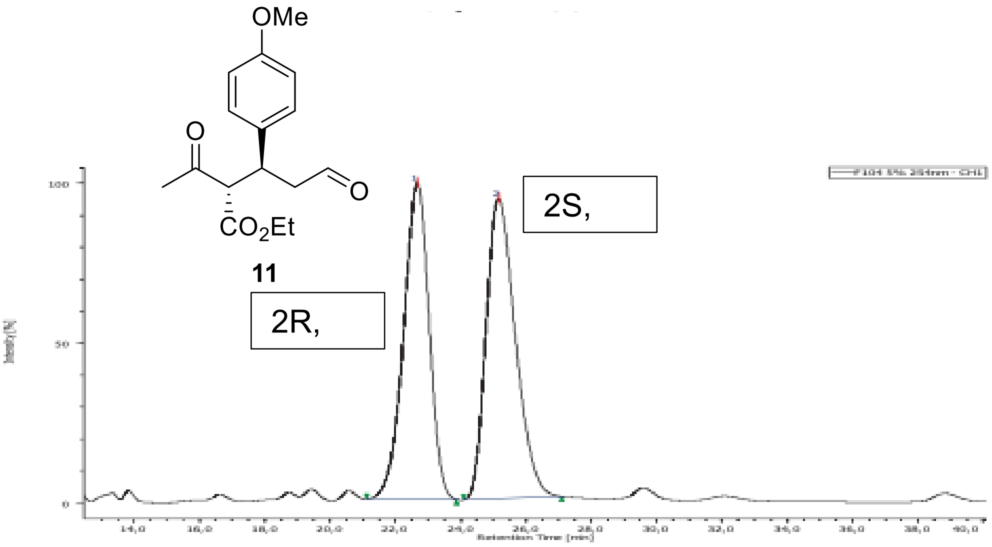

Peak	tR	Area	Height	Area%	Height%	Symmetry
1	22.658	11024311	202346	48.023	51.337	0.912
2	25.142	11932084	191809	51.977	48.663	1.304

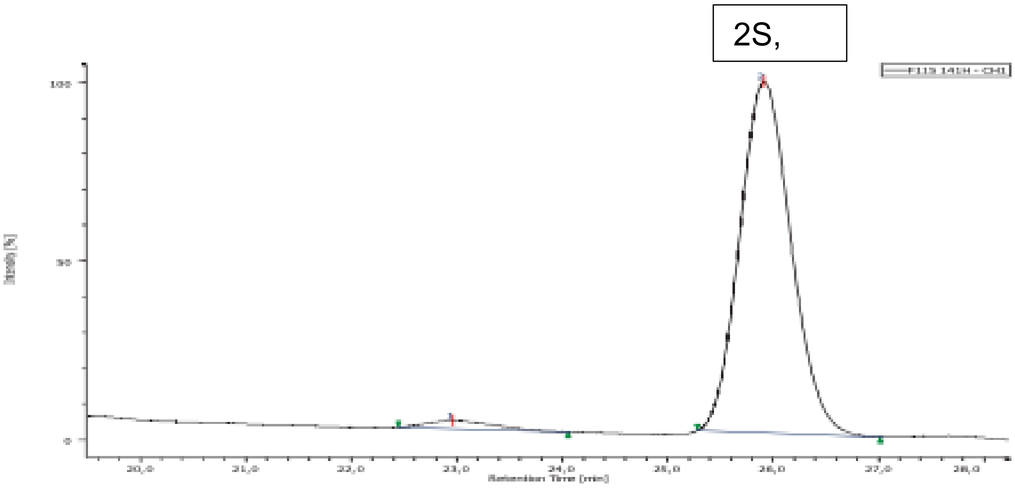

Peak	tR	Area	Height	Area%	Height%	Symmetry
1	22,967	5615	125	2,971	2,262	1,367
2	25,908	183384	5411	97,029	97,738	1,113

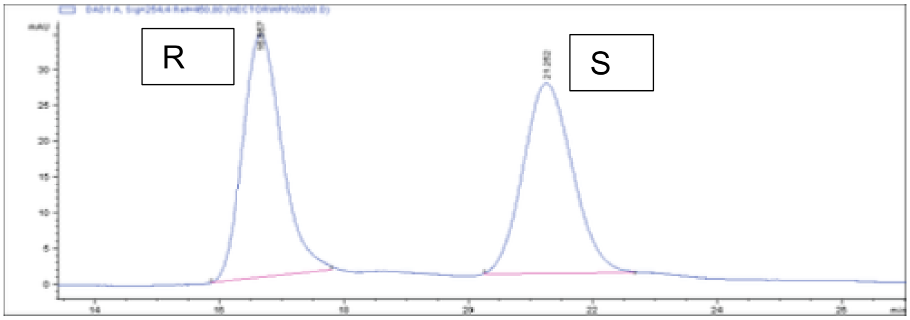

#	Time	Area	Height	Width	Area%	Symmetry
**1**	16.657	1485.3	34.2	0.5547	50.227	0.835
**2**	21.252	1471.8	26.6	0.7021	49.773	0.885

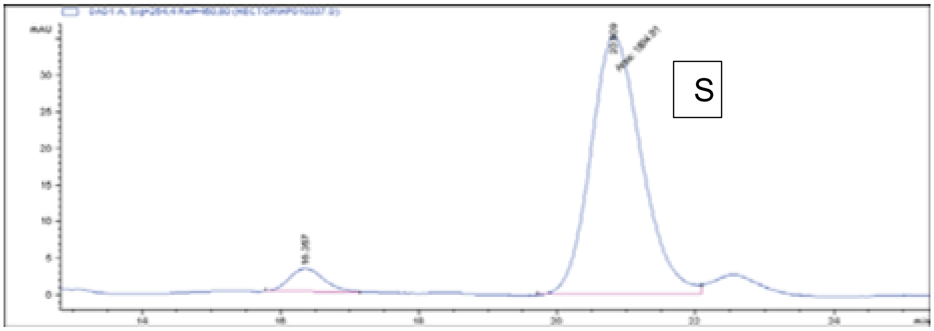

#	Time	Area	Height	Width	Area%	Symmetry
**1**	16.357	109	3.1	0.4196	5.696	0.871
**2**	20.809	1804	35.3	0.8519	94.304	0.791

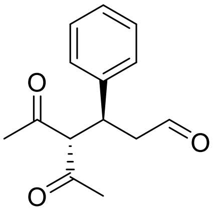

12

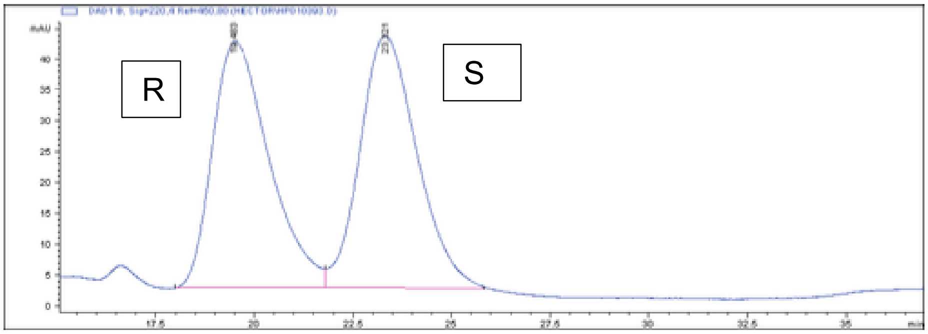

#	Time	Area	Height	Width	Area%	Symmetry
1	19.483	4004	40.1	1.1751	49.288	0.605
2	23.321	4119.7	41	1.1813	50.712	0.806

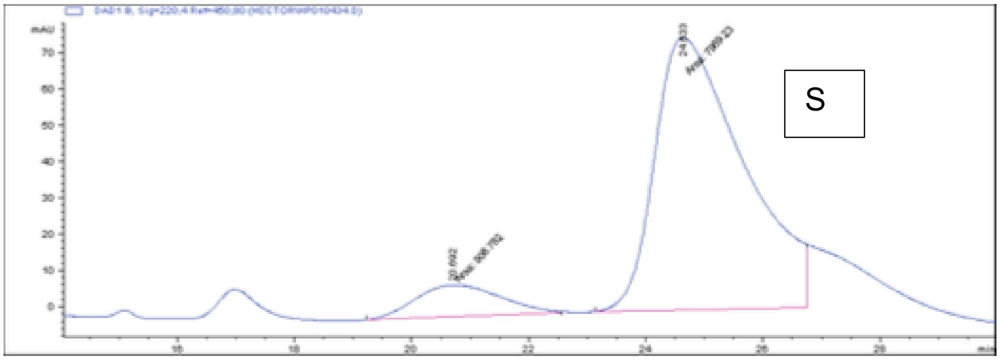

#	Time	Area	Height	Width	Area%	Symmetry
1	20.692	908.8	8.9	1.7091	10.236	0.779
2	24.633	7969.2	75.1	1.7679	89.764	0.467

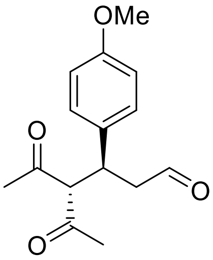

13

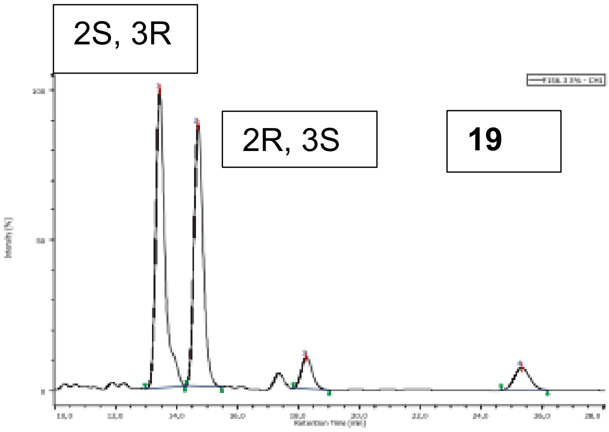

Peak Name	tR	Area	Height	Area%	Height%	NTP	Resolution	Symmetry Factor
anti	13,442	5231322	247772	47,231	48,576	11288	2,411	1,572
anti	14,700	4526485	217358	40,867	42,613	11839	5,910	1,219
syn	18,250	659154	26292	5,951	5,155	12077	8,835	1,158
syn	25,308	659057	18650	5,950	3,656	11654	N/A	1,108

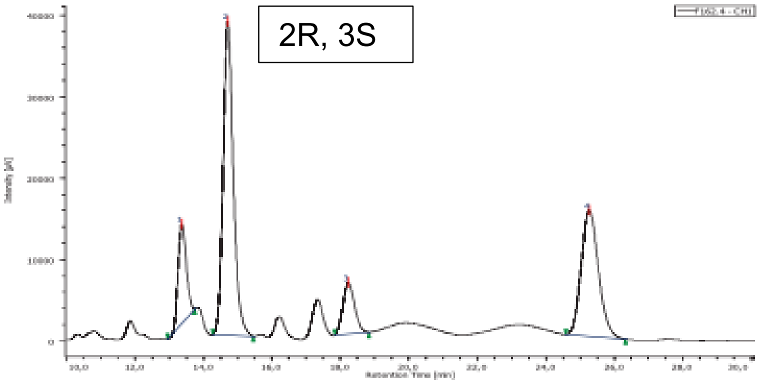

Peak Name	tR	Area	Height	Area%	Height%	Quantity	NTP	Resolution
anti	13,375	203245	12078	11,972	16,711	N/A	14038	2,719
anti	14,708	801278	38475	47,198	53,233	N/A	12246	6,022
syn	18,225	149660	6253	8,816	8,651	N/A	13002	9,031
syn	25,250	543496	15471	32,014	21,405	N/A	12039	N/A

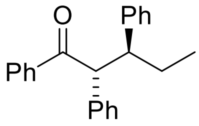

19
